# Factors associated with informal human milk sharing among donors and recipients: A mixed-methods systematic review

**DOI:** 10.1371/journal.pone.0299367

**Published:** 2024-03-08

**Authors:** Niamh Vickers, Anne Matthews, Gillian Paul

**Affiliations:** 1 School of Nursing, Psychotherapy and Community Health, Glasnevin Campus, Dublin City University, Dublin, Ireland; 2 School of Nursing, Midwifery and Health Systems, Health Sciences Centre, University College Dublin, Belfield, Dublin, Ireland; Landmark University, NIGERIA

## Abstract

**Background:**

The multiple benefits associated with the provision of human milk exceed individual health outcomes, engendering substantial economic, societal and environmental domains. Human milk is the absolute, unparalleled source of nutrition for infants. Informal human milk sharing is a modernistic and rapidly progressing practice. No systematic review of the factors associated with this contemporary practice among donors and recipients of informal human milk sharing exists.

**Aim:**

The aim of this review was to identify, evaluate, synthesize and integrate the evidence on the factors associated with informal human milk sharing among donors and recipients.

**Methods:**

A mixed methods systematic review was conducted according to the Joanna Briggs Institute methodological guidance utilizing a convergent integrated approach. The following databases were systematically searched: CINAHL, Scopus, Medline and Embase and Web of Science between inception to August 2023. A grey literature search was conducted using multiple techniques. This review followed the Preferred Reporting Items for Systematic Reviews and Meta-Analyses guidelines.

**Results:**

Twenty-four studies were included in this review. Ten integrated findings relating to the factors associated with informal human milk sharing among donors and recipients were identified. The four integrated findings pertaining to donors included: altruistic motivation and value, resistance to commercialization and overcoming inaccessibility, uniting digital and personal connectedness and lack of awareness and acceptance of informal human milk sharing in healthcare settings. The six integrated findings relating to recipients included: maternal or infant factors, superiority and advantageous impact of breastmilk, human milk bank influences, digital connections and transparency, healthcare professional facilitation of informal human milk sharing, and professional and logistical implications.

**Conclusion:**

This review highlighted a multitude of factors that motivate, facilitate and impede the practice of informal human milk sharing. Future research is required to explore these factors further within broader geographical locations to enhance the generalizability and rigor of the body of knowledge. Further studies should consider the exploration of the experiences and psychological impact of informal human milk sharing on donors and recipients. The provision of human milk to all infants is an imperative public health endeavor and thus positioning this as a key benchmark for research and practice is crucial.

## Introduction

Breastfeeding is widely accepted as a preeminent public health endeavor with advantageous outcomes in the optimization of child health and survival [1]. Globally, organizations recommend exclusive breastfeeding for the first six months of life and the continuation of breastfeeding along with complementary food until two years and beyond [2, 3]. The benefits of breastfeeding transcend individual outcomes, and comprise parallel positive outcomes within the societal, environmental and economic domains [4]. Breastfeeding is a focal component of the Sustainable Development Goals (SDGs) which are championed by all United Nations (UN) member states. The global targets of the SDGs intend to create a healthier, more sustainable world, through ending poverty, protecting the planet and the pursuit of harmony and prosperity for all [5, 6]. Human milk is a sophisticated and individually specific bodily fluid that demonstrates inherent variability and adaptability [4]. It is characterized by extensive individual variation in terms of its nutritional composition and the presence of bioactive components [7]. Accordingly, there is a distinct absence of a perfect substitute for human milk as various constituents are exclusively present in human milk from a lactating individual [8]. Therefore, human milk is the absolute, unequaled source of nutrition for infants [4].

On a global scale, the exclusive breastfeeding rates for infants aged 0–6 months stand at a suboptimal 44% [2], despite the multifaceted benefits associated with breastfeeding. Prominently, a discernable gap frequently emerges between the expressed intention to breastfeed and its implementation, as obstacles impeding breastfeeding transcend individual factors and encompass intricate social, cultural and political dimensions [9]. In instances where mothers own milk (MOM) is unavailable or insufficient, alternatives that have been recommended include, breastfeeding by another lactating woman, expressed milk from a human milk bank (HMB) or a breastmilk substitute such as commercial milk formula (CMF) [10]. There is a global consensus that MOM is the preferred source of nutrition for infants and if not available, pasteurized donor human milk from a HMB is considered the desirable choice [11–14].

The practice of human milk sharing is not a recent phenomenon but rather has origins in antiquity [4]. The act of sharing human milk can be attributed to the practice of ‘wet nursing’, wherein infants were breastfed by a lactating woman who was not their biological mother. The term wet nursing, or cross-nursing has been observed in various cultures worldwide spanning centuries [15]. The phenomenon of human milk sharing has progressed exponentially and the modern day banking of human milk originated in the 20^th^ century [16]. The availability of breast pumps, including high-quality double breast pumps and the capacity to store expressed human milk (HM) have categorically enhanced the viability and feasibility of HM sharing [17, 18]. Human milk banks (HMB) offer the distribution of pasteurized donor human milk (DHM) predominately to vulnerable infants when MOM is insufficient or unavailable [19, 20]. HMBs are instrumental in the provision of HM to infants, in particular preterm infants with vulnerabilities [20], however they are not universally available or widely accessible worldwide [21, 22]. Additionally, for profit HBMs have emerged, engaging in remunerating lactating women for their expressed human milk, which is subsequently sold to healthcare institutions [23].

The emergence of digital technologies has propelled the evolution of human milk sharing [24]. The concept of peer-to-peer human milk sharing is one such advancement in this practice and has been opined as a modern embodiment of an ancient practice [25]. The use of human milk through informal, peer-to-peer human milk sharing is increasing globally [26]. The recognition of commonly interchangeable terms used for this practice include: ‘*peer-to-peer milk sharing*,’, ‘*informal milk sharing*’, ‘*milk sharing via the interne*t’, ‘*internet based peer-to-peer milk sharing*’. The term informal human milk sharing (IHMS) will be used within this current review and pertains to the specified terms outlined above. These terms, used reciprocally, denote the practice of the voluntary and commerce-free trading of human milk outside the HMB system [18]. Inherent to this practice is the collaboration and agreement of donors and recipients of human milk in relation to the exchange [27]. Commercialization of human milk is becoming another recent advancement, where numerous internet-based platforms facilitate the commercial selling and purchasing of human milk [28].

In recent years, a growing body of research has been dedicated to exploring various aspects related to the practice of IHMS. Nevertheless, it is important to note that as of now, no systematic review specifically addressing this subject exists or is currently underway. Nonetheless, two recent scoping reviews by Akma Jamil et al. [24] and Kullmann et al. [29] have provided comprehensive mappings of the existing scholarly literature concerning IHMS, encompassing both the United States of America (USA) and International contexts, respectively. These prominent reviews in the realm of IHMS have acted as catalysts for enhancing the breadth and depth of understanding surrounding this contemporary practice [4]. Within the domain of IHMS, donors and recipients are the key actors inherent to this practice and it is crucial to gain an in-depth, and comprehensive understanding of the factors that motivate, facilitate and impede them to share or receive human milk. There is a justification for undertaking a systematic review to effectively integrate the existing evidence relating to these factors which will aim to assess consistency of findings across the literature, their generalizability to diverse settings and populations and potential implications for informing future healthcare decision-making [30, 31].

## Materials and methods

### Study aim and review questions

This MMSR aimed to identify, evaluate and integrate the quantitative, qualitative and mixed-method studies pertaining to the factors associated with informal human milk sharing among donors and recipients. The following questions aligned to the review question were:

•What are the factors (motivations, barriers and facilitators) associated with the practice of informal human milk sharing among donors?

•What are the factors (motivations, barriers and facilitators) associated with the practice of informal human milk sharing among recipients?

### Study design

This mixed methods systematic review (MMSR) was conducted in accordance with the Joanna Briggs Institute (JBI) guidance for conducting MMSRs as outlined in Chapter 8: Mixed methods systematic reviews in the JBI Manual for Evidence Synthesis [32, 33]. The protocol for this systematic review was registered on International Prospective Register of Systematic Review PROSPERO CRD42023405653 and published with HRB Open Research [4]. The protocol for this review has been approved following a peer review process. This has provided a robust foundation for the execution of this current MMSR. This review was completed in accordance with the Preferred Reporting Items for Systematic Reviews and Meta-analyses (PRIMSA) flow diagram [34]. This contemporary 27-item checklist was published in 2021 and was incorporated in this review as detailed in S1 File [34].

### Search strategy

A three-phase process was utilized for the search strategy as recommended by JBI and as documented in the approved protocol for this mixed-methods systematic review [4]. This search strategy was developed and conducted to find both published and unpublished studies. Foremost, a preliminary search of CINAHL (Cumulative Index to Nursing and Allied Health Literature) Complete, Scopus (Elsevier) and MEDLINE was undertaken incorporating key terms. An analysis of the key words/ terms in the titles, abstract and index terms was used to describe the articles yielded in the preliminary search which facilitated the development of a comprehensive search strategy to be developed. The keywords and search terms were peer-reviewed by the Subject Librarian in the School of Nursing, Psychotherapy and Community Health, Dublin City University. A systematic and iterative process utilizing synonyms, subject headings, Boolean operators (“AND” and “OR”) and controlled vocabulary terms were used for each database search. The full search strategy for each of the databases is detailed in S2 File. The following databases were searched: CINAHL (Cumulative Index to Nursing and Allied Health Literature) Complete (inception 1961), Scopus (inception 2004), MEDLINE (inception date 1879) and EMBASE (Excerpta Medica Database) (inception date 1980). The date of the final search was 14th August 2023. No language or date restrictions were applied to the searches. This was to ensure all relevant literature was identified and to facilitate a comprehensive search. In addition, a grey literature search was conducted using multiple techniques. First, Google Scholar was searched to supplement principal database resources to retrieve additional literature (including Grey literature). This was incorporated to improve the evidence base in retrieval of additional resources [35]. Google Scholar has been documented as a suitable supplementary resource for additional sources of evidence, including grey literature [36]. It has been suggested that grey literature yielded from Google Scholar appears after approximately 20 to 30 pages of the search results and thus a title screen of the first 60 pages (600 resources) was conducted [36]. Both title and full text search of Google Scholar was conducted. The rationale for this is founded on the affirmation that grey literature sources in the form of organizational reports and theses are predominately yielded via title searches [36]. The utilization of both title and full text searching was to ensure comprehensiveness and completeness. Additionally, a search of Web of Science and Open Grey was conducted to increase the breadth and depth of sources of evidence for the MMSR. Dissertation Abstracts was searched via the ProQuest database and a keyword search was conducted to source unpublished theses and dissertations relevant to the review question.

### Study selection

Inclusion criteria determined by using the PICo framework focused on studies exploring the factors (motivations, barriers, facilitators) of informal human milk sharing (Interest) from donors and recipients who engage in informal human milk sharing (Population) from a global perspective (Context) as recommended by JBI for MMSR [33]. Studies that also included other components of milk sharing such as wet nursing/cross feeding and milk banks were also included, if the practice of informal human milk sharing was also reported, but only data relating to informal human milk sharing were extracted for this MMSR. The PICo framework enabled the identification of the defining characteristics for the inclusion criteria of this MMSR as outlined in Table 1.

**Table 1:** PICo framework, inclusion and exclusion criteria of the MMSR.

**PICo**	Inclusion criteria:	Exclusion criteria:
**Population**	Studies including donors and recipients of informal human milk sharing	Studies exclusively investigating donor milk banks, commercial milk banks, or wet nursing/cross feeding. Studies investigating clinical outcomes of donor human milk. Studies exploring perspectives from individuals other than donors or recipients. Studies which are not primary research studies.
**Interest**	Studies on factors (motivations, barriers, facilitators) of informal human milk sharing	
**Context**	Globally	

**Fig 1:**
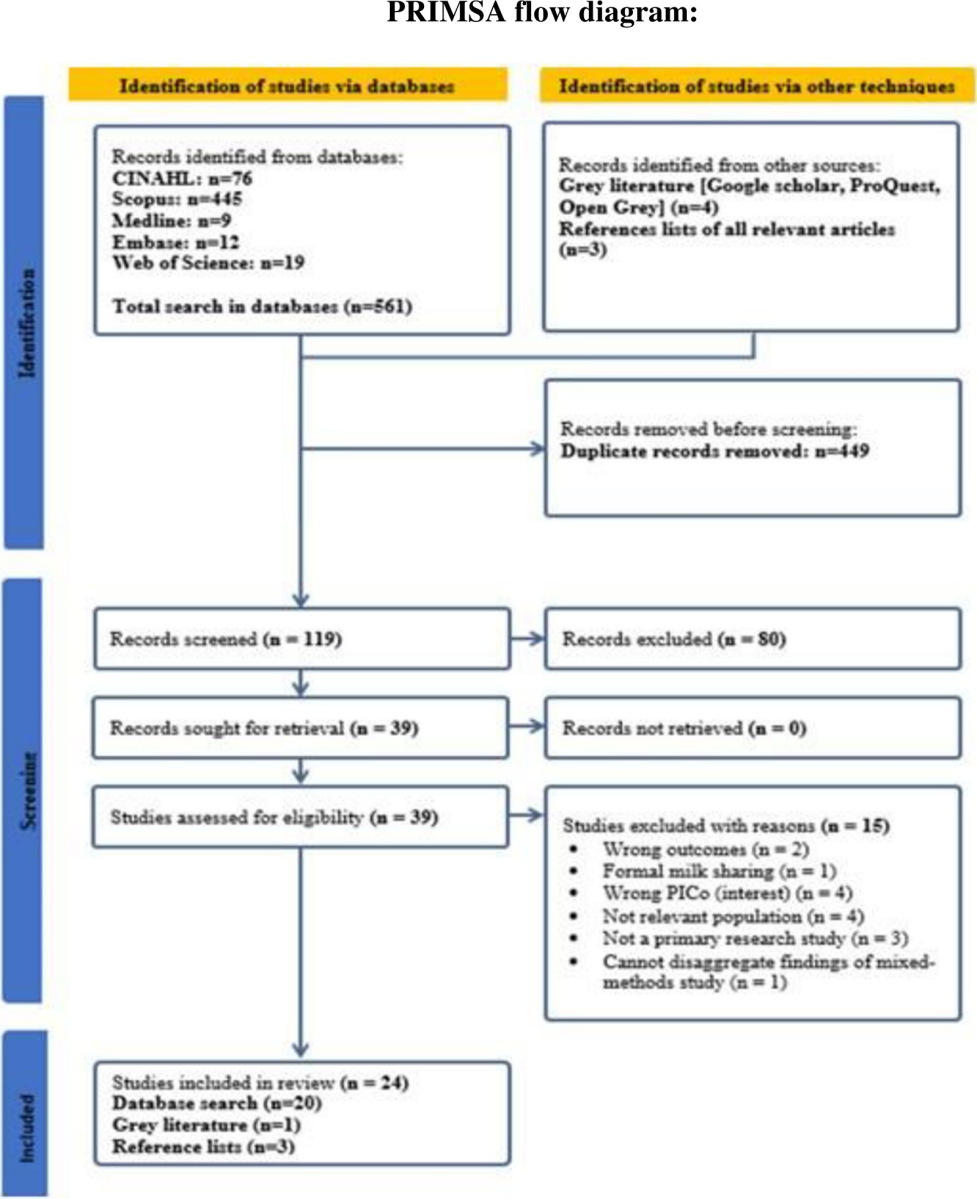
PRISMA flowchart of the study selection and inclusion processes.

The review considered original and grey literature sources with no language or date restrictions applied. The review included qualitative, quantitative and mixed-method studies. Mixed-method studies were considered only if the individual qualitative and quantitative components could be extracted separately [37]. In total, 568 citations were identified through the systematic search and were exported to Zotero Citation Management System. References were exported from Zotero Citation Management System version 6/2022 to Covidence and 449 duplicates were removed. Using Covidence, titles and abstracts were screened independently by the first author. A second co-author completed title and abstract screening independently. Following this, 39 full-text studies were screened and assessed independently by the authors for congruence with the specified inclusion criteria. Of these, 15 studies were excluded with explicit justification given for excluding. Through constructive discussions, any disagreements were resolved and a final decision was reached using a consensus-driven approach. The final review included 24 studies as presented in the PRIMSA flow diagram as visually presented in Fig 1.

### Risk of bias assessment (methodological quality)

A fundamental and key differentiating feature of a systematic review is the rigorous appraisal of the studies included in the review [38]. This key step in the systematic review process relates to the risk of bias assessment [39]. The terms critical appraisal, bias assessment, assessment of study validity, assessment of methodological limitations are terms used interchangeably in the literature to describe this process. The premise of this step is to evaluate the methodological quality of each separate study. All studies were assessed using the pertinent JBI critical appraisal tool dependent on the study design of each separate study. Eligible quantitative studies and the quantitative components of the mixed methods studies were evaluated with the relevant standardized JBI quantitative appraisal tool. Qualitative studies that were included and the qualitative components of the mixed-methods studies were evaluated using the standardized JBI qualitative critical appraisal tool. A key characteristic of the inclusion of mixed-methods studies was affirming that the qualitative and quantitative components could be extracted separately so that appropriate appraisal, extraction, synthesis and integration could be conducted. A quality percentage score was given to each criterion that the assessment tool evaluated [40]. Grey literature was appraised for methodological quality using the ACCODS checklist [41]. All studies, regardless of their methodological quality underwent data extraction and synthesis. The appraisal outcomes for each of the 24 included studies are included in S3 File. The most common criterions in the risk of bias assessment that led to a reduction in quality in the qualitative studies and qualitative components of the mixed studies was the exclusion of a statement locating the researcher culturally or theoretically, the exclusion of an explicit portrayal of the influence of the researcher(s) on the research and not stating the philosophical perspective of the research. In the quantitative studies and quantitative components of the mixed studies, the aspects of the risk of bias assessment that were most commonly omitted and subsequently led to a reduction in quality were the identification of confounding factors and strategies to deal with them.

### Data extraction

Data extraction was undertaken in Covidence using the standardized JBI data extraction form for MMSR following a convergent integrated approach [32]. This is the recommended data extraction for MMSRs following a convergent integrated approach [37]. Data extraction was completed by the first author and the process was cross-checked by the co-authors to ensure accuracy. The following information was extracted and tabulated for each study: author, year, study design, methodology, number and characteristics of participants, study setting and any other context information, geographic location, outcomes or findings significant to the review objectives.

### Data transformation, synthesis and integration

Following the data extraction phase, the quantitative data underwent a transformative process referred to as ‘qualitization’. This technique involved the transformation of quantitative data into textual descriptions and narrative interpretation, which enabled a fluid integration with the data derived from the qualitative studies, so as to respond directly to the review questions. The process of qualitization is recommended by the JBI methodology group, as it reduces the potential errors of converting qualitative data into numerical format, termed quantitization [33].

A convergent integrated approach according to the JBI methodology for MMSRs was applied in this review. Subsequently, the qualitized data was assembled and then pooled together with the qualitative data from the qualitative studies and the qualitative components of the mixed methods studies, to identify categories based on similarity in meaning and to create a set of integrated findings in the form of line of action statements.

## Results

### Description of included studies

The characteristics of the final studies included in this MMSR are presented in S1 Table. Of the 24 included studies, four were quantitative (16.6%), three were mixed methods (12.5%), and seventeen were qualitative (70.8%), published between 2013–2022. The sample sizes ranged from 1 to 1050 participants with six studies focused solely on donors, four on recipients and fourteen 14 included both donors and recipients in their study. The majority of studies were conducted and based in the United States of America (n = 18) [17, 25, 42–57], two were conducted in Australia [16, 58], and one each conducted in Ghana, Canada, Turkey and the United Kingdom [15, 59–61]. While the primary location of the country in which the study was conducted was noted, some studies included some participants outside of their primary location and is evidenced in S1 Table [16, 25, 42, 43, 48–51, 58]. These combined additions included some participants from India, Indonesia, Columbia, Lebanon, Netherlands, Europe, Oceania, Asia.

### Review findings

This MMSR explored the motivations, barriers and enablers of donors and recipients associated with IHMS. The findings of this MMSR are discussed across the themes and integrated findings below and the donor and recipient cohorts are presented separately for clarity.

**Fig 2:**
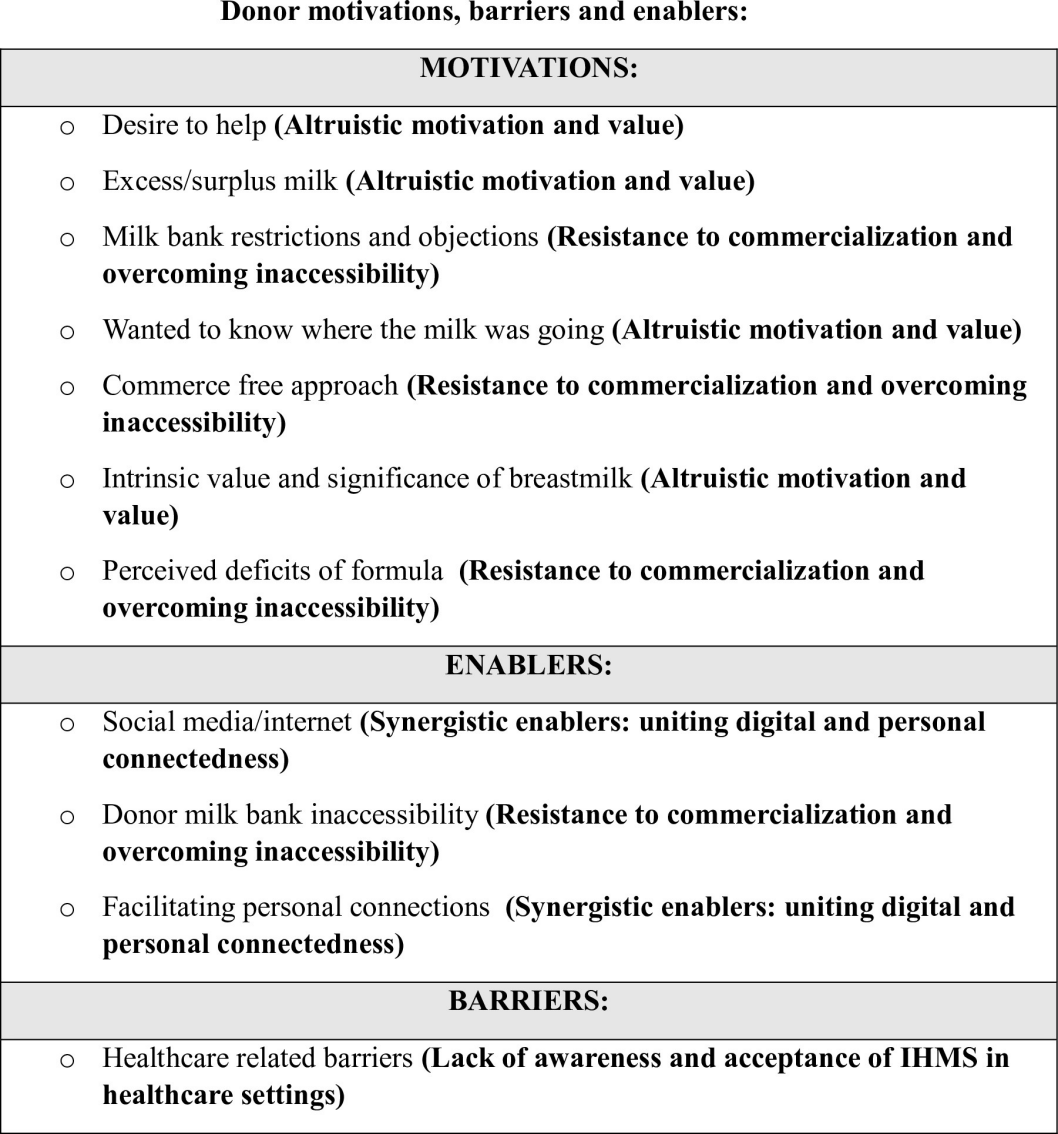
Categories and integrated findings for donor motivations, enablers, and barriers of IHMS.

**Fig 3:**
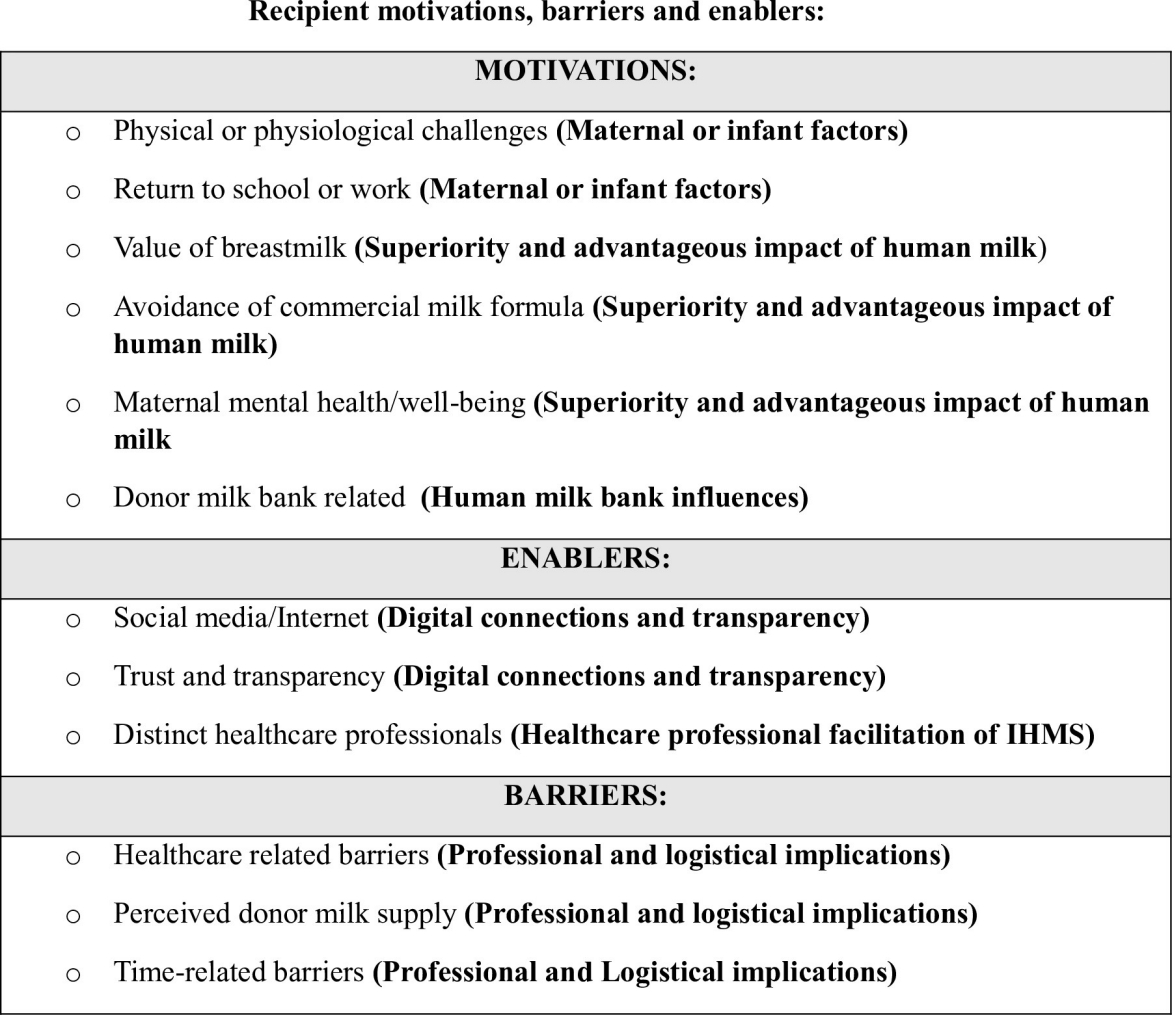
Categories and integrated findings for recipient motivations, enablers and barriers of IHMS.

In relation to the motivations, barriers and enablers, of donors and recipients associated with IHMS, the qualitized data, qualitative data and emergent categories and integrated findings are detailed in S4 and S5 Files. These are summarized and visually presented in Figs 2 and 3.

The findings of the MMSR relating to donor participants motivations, enablers, and barriers included four integrated findings which are presented in Table 2 and discussed below.

**Table 2:** Integrated findings for donor motivations, enablers, and barriers of IHMS.

1. Altruistic motivation and value
2. Resistance to commercialization and overcoming inaccessibility
3. Synergistic enablers; uniting digital and personal connectedness
4. Lack of awareness and acceptance of IHMS in healthcare settings

#### Altruistic motivation and value

The desire to help was the most significant motivator reported for donors to engage in the practice of IHMS and was reported in eleven of the included studies [16, 25, 45–48, 53, 58–61].This was often underpinned by the donors reporting a surplus or excess of breastmilk [16, 17, 46–48, 60, 61]. The beliefs about/ of the significance and exceptional value of breastmilk held by donors was evident in six of the studies within the review [45–48, 58, 60]. Breastmilk was perceived as ‘powerful’ [56, 60], ‘precious’ [48] and donors expressed a profound reluctance to waste it, thus this formed a compelling reason to engage in IHMS [48, 58]. The substantive benefits of breastmilk were noted by donors including the importance of breastmilk to infants [47, 48, 60] and mothers [60].The value of breastmilk perceived by donors in the review was evident in their motivation to seek information about the intended recipients which served as a stimulus for engaging in IHMS [25, 45, 61]. In four of the studies included in the review, donors performed a needs assessment on the intended recipients to ascertain why the milk was required and where the milk was going [25, 51, 60, 61].

#### Resistance to commercialization and overcoming inaccessibility

Donors articulated the superiority of DHM to CMF which they noted enables infants to attain all their nutritional and vitamin requirements [47], thrive, and experience ‘*none of that discomfort*’ associated with CMF [25]. Various personal objections and restrictions associated with donor milk banks (DMB) were evident within the review which served as motivator for engaging in IHMS. These included the dissatisfaction with the exorbitant costs for recipients in obtaining DM from a DMB [45, 51], the impracticalities associated with DMBs such as logistical efforts or the various regulations associated with donating to a DMB [45, 48, 51, 60]. In one study it was noted that most donors could not donate to the DMB as they did not pass the pre-donation screening [61]. Similarly, inaccessibility to donate to DMB’s was noted in a further study where donors met various challenges with donating such the lack of DMBs in their locality, DMBs were not accepting donations, they did not meet the requirements of donating or a lack of response from the DMB [51].These prohibitions emerged as enabling and motivating factors for donors to participate in IHMS. Additionally, some donors were propelled to engage IHMS rather than a DMB as they preferred that their milk remained unpasteurized, as they believed this retained the distinct and valuable properties of BM [45, 51]. Various studies identified that donors were compelled to IHMS as they preferred a commerce-free approach associated with most IHMS arrangements [45, 51]. In their study, Onat and Karakoç (2019) identified that all donors in their study (n = 48) received no payment for the milk exchange, emphasizing the prevailing inclination of donors towards a commerce-free approach, aligning with the philosophy of these other findings [61].

#### Synergistic enablers: Uniting digital and personal connectedness

Digital platforms were a common enabling factor for donors to engage with IHMS. In the review, only one of the studies specifically reported on IHMS that did not occur via online platforms [53]. In their study, Obeng et al. (2022) did not specify if online platforms were used amongst any donors in their study and within the presentation of their findings, this was not evident [59]. In another study, milk sharing devoid of the use of digital platforms was implicit [58]. In the other 17 studies included in the review which studied donors, the sharing by donors of human milk via online networks was frequent. Within four of the reviewed studies, the internet and social media platforms were explicitly identified as predominant facilitators and enablers for donors to engage in IHMS [25, 45, 46, 60]. In two of the studies, donors became aware of IHMS through self-initiated internet searches which served as a significant enabling factor to engage in the practice of IHMS [45, 60]. The utilization of digital platforms in initiating the practice of IHMS served as a means of enabling the continuation of IHMS and the development of ongoing personal connections among the donors and recipients [46, 60]. In their study, O’ Sullivan *et al*., (2018) found that most donors provided milk to a friend or someone they knew [52]. In this current review, it was noted that donors often articulated that they judged the needs and circumstances of the requesting recipients which provided assurance that their milk was being used for a genuine need [25, 51, 60]. The personalization of the exchange is evidenced by the majority of donors and recipients meeting in person for the exchange of DM [25, 51, 57, 60, 61].

#### Lack of awareness and acceptance of IHMS in healthcare settings

In parallel to the evidence ascertaining that donors engage in IHMS independently, it is also observed that often milk sharing by donors is practiced without considerations or input from healthcare professionals, which is a notable barrier. The majority of donor participants in one study indicated that they were not provided with any guidance or insight regarding options for milk sharing by healthcare professionals (HCP) [45]. This finding was reinforced by Onat and Karakoç (2019), where it was ascertained that the majority of donors reported that healthcare workers did not recommend milk donation [61]. Two of the studies reported that due to the unregulated nature of IHMS, donor participants often felt they were doing something wrong or illegal [46, 60]. In three of the studies, it was ascertained that certain healthcare professionals such as lactation consultants and midwives were sometimes supportive of the practice [17, 45, 60] and acted as facilitators in the milk exchange process [17].

The findings of the MMSR relating to recipient participants motivations, enablers and barriers and six integrated findings which are presented in Table 3 and discussed below.

**Table 3:** Integrated findings for recipient motivations, enablers and barriers and of IHMS.

1. Maternal or infant factors
2. Superiority and advantageous impact of breastmilk
3. Human milk bank influences
4. Digital connections and transparency
5. Healthcare professional facilitation of IHMS
6. Professional and logistical implications

#### Maternal or infant factors

In this review, physical or physiological factors relating to the mother or infant were primary motivators for recipients to source donor milk. Maternal milk insufficiently was noted in 11 studies as the most frequently cited maternal-associated factor prompting the exploration and engagement in the practice of IHMS [13, 16, 17, 43, 44, 49, 56, 58, 60, 61] Two studies compiled data on the proportion of participants who experienced insufficient milk supply. 68% and 88% of participants in studies by Schafer *et al*., (2018) and Onat and Karakoç (2019) respectively, reported maternal milk insufficiency [56, 61]. Infant related issues were also frequently cited as influential factors to engage in IHMS [17, 43, 44, 49, 54, 57, 58]. In one study, more than half (57.8%) of participants articulated infant related issues as the reason for seeking donor milk [57]. Some of the studies reported on explicit infant issues which motivated participants to consider IHMS, including the most commonly cited infant related issues which were: weight concerns [49, 44], breastfeeding attachment issues [17, 43, 49, 58], health related problems with the infant [43, 54, 58] and history of prematurity [17, 54]. Another motivating factor for engaging in IHMS was the return to employment or education which was identified in two of the studies in the review [48, 54].

#### Superiority and advantageous impact of breastmilk

In the review, it was noted that interrelated factors pertaining to the perception of breastmilk, CMF and the impact of donor milk on wellbeing formed significant motivators for the engagement in IHMS. In eight studies breastmilk was consistently characterized by recipients as beneficial, healthy and the best alternative for infant feeding in the absence of MOM [13, 43, 44, 48, 55, 60, 61]. One study reported that 83.3% of recipients chose donor milk as it was the healthiest option [55]. Further, Onat and Karakoç (2019) reported that 62.5% of recipients viewed donor milk as the most natural way to feed infants in the absence of MOM [61]. Commercial milk formula was consistently noted as being an inferior alternative choice of infant feeding within the review [13, 16, 43, 48, 50, 55, 60, 61]. The motivation to avoid CMF due to reported undesirable side effects for infants was noted among recipients, these included pain [13, 48], constipation [13, 44], allergy [43, 44, 48, 53]. Three of the studies also explicitly highlighted recipients’ concerns with risks associated with CMF and avoidance of these was a clear justification for engaging in IHMS [50, 55, 61]. In four of the studies, it was identified that the risks associated with CMF were greater than the risks of providing donor milk to infants [50, 55, 58, 60]. Concerns regarding the constituents of CMF was another notable factor in choosing IHMS in some of the studies [43, 49, 60]. The impact of CMF on stress and maternal well-being was reported in some of the studies. In one study, it was identified that having to supplement with CMF resulted in negative emotions [48]. The actual or perceived use of CMF resulted in feelings of depression, stress or anxiety in recipients in two of the studies [13, 48]. Corresponding to this, receiving donor milk resulted in the alleviation of stress and provision of comfort in several of the included studies [13, 43, 56]. None of the included studies reported that IHMS resulted in negative emotions. In their study of IHMS recipients (n = 205), Schafer *et al*., (2018) highlighted a significant positive emotional associate with IHMS [56]. In another study, an analysis of recipients satisfaction with their supplement choice reported that recipients of DHM were more satisfied with their supplement choice compared to those who chose CMF as a supplement [55].

#### Human milk bank influences

In the review, DMB were discerned as being a motivating factor for recipients to engage with IHMS for several reasons. It was noted in some of the studies, that recipients would not be eligible to receive DHM from a DMB due to not meeting specified criteria [42, 48, 51]. Three of the studies identified the prohibitive costs of DMB’s which presented financial obstacles to the recipients in being able to engage or sustain the ongoing costs, which compelled them to engage with IHMS [42, 44, 51]. The inaccessibility to DMB due to proximity was also documented as motivator to engage in IHMS [42, 51]. Some studies also reported on personal beliefs held by some recipients regarding DMB which propelled them to the alternative of milk exchanging via IHMS. These included the dissatisfaction with DMB pasteurization which was identified in one study by recipients as a process which diminishes valuable proteins [42] and a recipient belief of the avoidance of anything overly interventionist, a sentiment that correlates with DMB [47].

#### Digital connections and transparency

The use of digital platforms and the subsequent interactions with donors through communication, open dialogue and connection, founded primarily online, were noticeable enabling factors in the engagement of IHMS for recipients in this review. As previously noted, only one of the studies specifically reported on milk exchanges that did not occur via online platforms [53] and milk sharing devoid of the use of digital platforms was implicit in a further study [58]. The remaining studies in the review reported that establishment of milk exchanges online was frequent. Three of the studies reported that social media made the practice of IHMS more accessible to participants [13, 50, 60]. It was noted in two studies that strong social support for recipients was predominantly via online platforms which emphasizes the enabling role of digital connection in the practice of IHMS[13, 56]. The establishment of trust, derived from transparent discourses surrounding donors and the construction of personal connections between the recipients and donors were noted as enabling factors in the engagement and continuation of IHMS [13, 16, 25, 50, 60]. Some of the studies documented the concept of donor screening by recipients, indicating that informal or lay screening methods were used rather than a formal screening process, prior to the acceptance of DM in seven of the studies [13, 16, 43, 50, 58, 60, 61]. The most frequently reported areas of lay screening related to donors use of alcohol [50, 13, 60, 61] medications [13, 42, 50, 60, 61] and smoking [13, 42, 60, 61]. In two of the studies, it was reported that open communication between the donors and recipients provided a sense of reassurance and comfort to the recipients and was a pivotal factor for engagement with IHMS [13, 50]. This finding is supported by reports of the rejection of donor milk in instances where there was difficulty communicating with the donor or where a personal connection with the donor did not develop [13, 50]. Additionally, comfort in the use of DHM was also derived from knowing that the donor was also breastfeeding their own child and was reported in three of the studies [47, 44, 60]. Notably, a rejection of donor milk was also noted if donors were not breastfeeding their own child [50].

#### Healthcare professional facilitation of IHMS

In the review, four studies reported that certain HCPs were an enabling factor in the participation in IHMS [17, 42, 44, 55]. The most cited healthcare professionals associated with enabling IHMS identified in this review were breastfeeding specialists/lactation consultants [42, 44], midwives [17, 42] nurses [42] and medical professionals [42, 55]. While some studies reported HCPs as enabling factors in IHMS, this finding was not consistent and will be discussed further in this review.

#### Professional and logistical implications

Professional and logistical implications of IHMS were noted as barriers within the current review. Foremost, HCP related barriers were evident. While some HCPs were cited as being enablers of milk sharing as discussed above, this finding was in contrast with some studies which noted they were a barrier to the engagement of IHMS. Notably, in their study, Cassar-Uhl and Liberatos (2018) some HCPs were identified as the source of the DHM however this category was identified as the lowest scoring category overall, with only one in five recipients reporting their source of DHM was identified through HCPs [55]. This is implicit of notable professional barriers to IHMS. In their study, McCloskey and Karandikar (2018) noted that many recipients acknowledged an awareness of the formal and informal professional prohibitions relating to promoting and permitting of IHMS by organizations and HCPs [42]. In a further study, a lack of awareness and exhibited opposition towards the concept of IHMS by healthcare professionals was noted [44]. Another key logistical barrier which was reported in four studies in the review, was the fear or actual experience of running low on DHM [13, 25, 43, 44]. Another significant barrier noted in the review pertaining to recipients was a time related barrier where the logistical efforts of attaining DHM was time-intensive in terms of the geographical distance of donors from the recipients [42–44, 60].

## Discussion

This is the first MMSR to synthesise the evidence on the motivations, barriers and facilitators of donors and recipients of IHMS. In this MMSR, twenty-four studies met the inclusion criteria. The key findings of this review detail an array of factors associated with IHMS from the key actors involved in this emerging, and rapidly advancing practice. This review has elucidated the intrinsic and absolute value of breastmilk, positioned by both donors and recipients. The profound significance and superiority ascribed to human milk by both groups underscores its unique and highly positioned standing which formed a key motivator for the engagement in IHMS. Analogous to this, is the commonality of the perceived deficits of CMF compared to HM within both donor and recipient cohorts. This propelled donors and recipients to engage in the sharing of HM. These findings of the perceived intrinsic superior value of HM and the inferiority of CMF is evidenced in further empirical research including an evaluation of parents perceived healthiness of MOM, peer-shared HM and CMF, where it was consistently noted that CMF was the least reported healthiest option for infants [62]. Interestingly, HMB was identified as a motivator for both groups to engage in IHMS and as an enabler for donors to share milk as opposed to donating to a HMB. Human milk banks were multifactorial in relation to the reasons why both groups engaged with IHMS including; the inaccessibility for many recipients and donors to engage with HMB due to restrictions, prohibitions, financial and logistical implications. Consequently, these factors formed reasons for the engagement in IHMS. Interestingly, these notable restrictions and prohibitions while acting as motivators to the engagement in IHMS in this present study also act as known barriers to the donation of milk to HMB in the domains of individual, systemic and social barriers, as identified in a recent systematic review [63]. It is widely acknowledged that HMBs are not widely accessible, nor is DHM universally available to all infants [20, 21, 64]. The absence of adequate infrastructure, regulation and guidance within HMBs poses challenges to ensuring quality control, safety provisions and scalability [65]. In contrast to many jurisdictions, Brazil has the most comprehensive, publicly funded network of HMBs in the world [66]. There is a need for policy makers and governmental bodies to provide guidance and explicit recommendations for HMB and the provision of DHM to infants in the absence of MOM. As such, there is a demand for comprehensive global guidelines outlining the essential standard operating procedures necessary for the effective operating of HMBs [67].

A prominent driver for donors in the engagement in IHMS was their unreserved motivation to assist and help others. This altruistic motivation is intertwined with the utmost, significant value perceived by donors of HM, whereby they wanted to know where their milk was going, ensuring a genuine need. This unequivocally relates to the recipient enabler for IHMS, where trust and transparency were key enablers for the engagement with the practice of IHMS. The uniting of digital technologies and subsequent personal connectedness, stemming from online platforms, was a synergistic enabler which led to a cooperative and shared understanding between donors and recipients. This shared connection fostered trust and transparency which were requisite factors in the consideration of engagement with IHMS for both donors and recipients. The most pervasive barrier to IHMS were healthcare related barriers for both the donor and recipient groups. This was evidenced by a lack of input from HCPs in IHMS, limited guidance/insight to the practice or an opposition to milk sharing held by HCPs. This may be attributed to global health organizations who discourage the use of informal peer milk sharing such as the American Academy of Paediatrics [3] and the United States Food and Drugs Administration [68]. This opposition to the practice is due to the inherent possible risks with the sharing of unpasteurized human milk, a complex bodily fluid, in the transmission of disease, contamination when expressing or handling or indeed contamination with various, potentially unsafe substances [69–71]. Somewhat differing, the World Health Organization in the Global Strategy for Infant and Young Child Feeding emphasize that where breastfeeding is not feasible, the most suitable alternatives in a hierarchical depiction, is expressed MOM, human milk from a healthy wet-nurse or human-milk bank, or an appropriate breastmilk substitute is advisable [10]. The Academy of Breastfeeding Medicine (ABM)in their position statement on the sharing of human milk provide guidance relating to milk sharing, incorporating key strategies which should be implemented to maximize the safety of milk sharing including: health screening of the donor and safe milk handling practices [72]. However, this position statement refutes the engagement of internet-based human milk sharing in any eventuality [72]. It has been articulated that HCPs possess the potential to endorse regulations concerning milk sharing while actively participating in collaborative decision making processes to inform and educate families about donor health screening and safe handling practices [29]. An interesting finding in this review was that some HCPs were noted as being facilitators of IHMS in a limited number of studies, this is in contract with most studies who explored this component and documented the lack of awareness and insight by HCPs in relation to IHMS. This warrants further investigation and exploration. This review noted that maternal factors such as actual or perceived low milk supply was noted as the most prominent motivator for the engagement in IHMS by recipients. According to estimates, a relatively small proportion of women are physiologically unable to breastfeed, however a larger proportion of lactating women express concerns relating to their milk production/supply [73]. The most commonly cited breastfeeding challenge and reason for early cessation of breastfeeding or reduced breastfeeding exclusivity is perceived milk insufficiency [74–77]. Breastfeeding challenges and the early cessation of breastfeeding is consistently reported as a significant, mediating factor for poorer maternal mood in the postpartum period [78–83]. Within this current review, the actual or perceived use of CMF was found to be associated with increased anxiety, stress and self-reported depression among recipients. Interestingly, it has been confirmed that exclusive breastfeeding is associated with a reduced risk for postpartum depression in a recent systematic review [84]. Notably, engaging in the practice of IHMS within this current review was aligned to positive feelings, a reduction in reported stress and in two of the studies, an articulated protective factor for the development of postnatal depression in recipients. This warrants further in-depth exploration to investigate the impact of IHMS on psychological well-being of recipient cohorts. This is necessitated by the verity that postpartum depression (PPD) is a widespread occurrence, with possible detrimental outcomes and significant estimates of one on five women experiencing PPD in the postnatal period globally [85].

## Strengths and limitations of the study

This MMSR utilized multiple reviewers in the conduct of the review process, including the screening, selection, risk of bias assessment, extraction, synthesis and integration. This enabled an explicit development of rigor and reliability within. Another key strength is the integration of qualitative, quantitative and mixed-methods studies in the review, following a convergent integrated approach, guided by the JBI methodology [86]. The incorporation of the standardized JBI critical appraisal tools for the risk of methodological quality/bias assessment was an explicit strength. The inclusion of all studies, irrespective of language or devoid of time limitations was an inherent strength, as well as the incorporation of grey literature sources in the included sources. Most of the studies in the MMSR included a single form of data collection at one time point which could potentially miss variability of changes in variables over time. Also, most of the studies included in this review were conducted in a limited number of countries, which may introduce geographic bias and reduce the generalizability of the findings to a broader population or different contexts within different countries or regions.

## Conclusions

This MMSR has provided an explicit interpretation and nuanced understanding of the motivations, facilitators and barriers of the key actors within the practice of IHMS. This comprehensive review has enabled a detailed understanding, synthesis and integration of the predominant factors associated with this modernistic practice including why and how donors and recipients are motivated, enabled and impeded in the practice of IHMS. Many of the studies identified in the MMSR were limited to a small number of countries and future research exploring the factors associated with IHMS is required in other countries and jurisdictions is warranted to add to the current body of knowledge in this area. This will enable more generalizable findings across wider geographical settings and cultural contexts. Such knowledge will further enable a greater understanding from a broader, all-encompassing perspective. The impact of IHMS on enhanced maternal psychological wellbeing was a finding in three of the studies where the receipt of DM by an informal means resulted in the alleviation of stress and the provision of comfort [13, 43, 56]. There is an imminent need to explore this further to leverage a more explicit insight and elucidate the potential influence of IHMS on the psychological wellbeing of mothers. Additional research is also warranted to investigate the experiences of individuals who engage in IHMS. Most of the studies were a cross sectional design and there is a need to explore the practice of IHMS at different time points. This will enable a richer and absolute exploration of the practice and will facilitate clarity on variability in factors and perspectives over time. The approach to infant feeding has enduring consequences on individual, societal and environmental health and sustainability [87]. Consequently, there is an imminent need for positioning the provision of human milk to all infants as a global priority.

## Supporting information

S1 File PRISMA checklist. (DOCX)

S2 File Search strategy. (DOCX)

S3 File Risk of bias assessment. (DOCX)

S4 File Donor synthesis integration qualitization file. (DOCX)

S5 File Recipient synthesis integration qualitization file. (DOCX)

S1 Table Characteristics of included studies. (DOCX)
