# Supplementary material for: Factors associated with informal human milk sharing among donors and recipients: A mixed-methods systematic review
Source: PLoS One. 2024 Mar 8;19(3):e0299367. doi: 10.1371/journal.pone.0299367 (PMC10923476; doi:10.1371/journal.pone.0299367)
Supplement: S2 File — (DOCX) [file pone.0299367.s002.docx]

**S2. File Search strategy:**

**CINAHL (Cumulative Index to Nursing and Allied Health Literature)**

| **Searches** | **Number of hits** |
| --- | --- |
| 1. (MH “Milk, Human+”) | 7679 |
| 1. breastmilk | 5499 |
| 1. Mothers milk | 5951 |
| 1. 1 OR 2 OR 3 | 9091 |
| 1. Peer sharing | 276 |
| 1. Peer-to-peer | 2127 |
| 1. Informal sharing | 91 |
| 1. Unscreened sharing | 3 |
| 1. Unregulated sharing | 3 |
| 1. “breastmilk donation” | 5 |
| 1. 5 OR 6 OR 7 OR 8 OR 9 | 2,450 |
| 1. Practices | 776,522 |
| 1. Perceptions | 189,701 |
| 1. Perspectives | 166,983 |
| 1. Views | 116,435 |
| 1. Attitudes | 396,864 |
| 1. Opinion | 52,003 |
| 1. Understanding | 247,312 |
| 1. Experience | 447,444 |
| 1. Motivations | 64,097 |
| 1. Barriers | 119,725 |
| 1. Facilitators | 19,402 |
| 1. 12 OR 13 OR 14 OR 15 OR 16 OR 17 OR 18 OR 19 OR 20 OR 21 OR 22 | 1,610,024 |
| 1. (MH “Internet”) | 53,210 |
| 1. Online | 105,867 |
| 1. Internet-based | 5,938 |
| 1. Interne* | 83,490 |
| 1. Communities | 369,098 |
| 1. Worldwide | 54,891 |
| 1. 24 OR 25 OR 26 OR 27 OR 28 OR 29 | 168,931 |
| 1. 4 AND 11 | 35 |
| 1. 4 AND 11 AND 23 | 26 |
| 1. 4 AND 11 AND 23 AND 30 | 15 |
|  |  |

**Scopus (via Elsevier)**

| **Searches** | **Number of hits** |
| --- | --- |
| S1. Human milk OR breastmilk OR mothers milk    TITLE-ABS-KEY (“human milk” OR “breastmilk” OR “mothers milk”) | 6,955 |
| S2. Peer sharing OR Peer-to-peer OR informal sharing OR breastmilk donation OR breastmilk donor OR unscreened sharing OR unregulated sharing  TITLE-ABS-KEY (“Peer sharing” OR “Peer-to-peer” OR “informal sharing” OR breastmilk donation” OR “breastmilk donor OR “unscreened sharing” OR “unregulated sharing”) | 190 |
| S3. Practices OR perceptions OR perspectives OR views OR opinion OR understanding OR experience OR motivations OR barriers OR facilitators  TITLE-ABS-KEY (Practices OR perceptions OR perspectives OR views OR opinion OR understanding OR experience OR motivations OR barriers OR facilitators) | 32,468,402 |
| S4. Internet OR online OR internet-based OR interne* OR communit* OR worldwide  TITLE-ABS-KEY (Internet OR online OR “internet-based” OR interne*) | 12,645,916 |
| S4. S1 AND S2  TITLE-ABS-KEY (“human milk” OR “breastmilk” OR “mothers milk”) AND (“Peer sharing” OR “Peer-to-peer” OR “informal sharing” OR breastmilk donation” OR “breastmilk donor” OR “unscreened sharing” OR “unregulated sharing”) | 157 |
| S5. S1 AND S2 AND S3  TITLE-ABS-KEY (“human milk” OR “breastmilk” OR “mothers milk”) AND (“Peer sharing” OR “Peer-to-peer” OR “informal sharing” OR breastmilk donation” OR “breastmilk donor” OR “unscreened sharing” OR “unregulated sharing”) AND (Practices OR perceptions OR perspectives OR views OR opinion OR understanding OR experience OR motivations OR barriers OR facilitators) | 153 |
| S6. S1 AND S2 AND S3 AND 4  TITLE-ABS-KEY (“human milk” OR “breastmilk” OR “mothers milk”) AND (“Peer sharing” OR “Peer-to-peer” OR “informal sharing” OR breastmilk donation” OR “breastmilk donor” OR “unscreened sharing” OR “unregulated sharing”) AND (Practices OR perceptions OR perspectives OR views OR opinion OR understanding OR experience OR motivations OR barriers OR facilitators) AND (Internet OR online OR “internet-based” OR interne* OR communit* OR worldwide) | 135 |

**Medline (via Pubmed)**

| #1 | "Milk, Human"[Mesh] OR “breastmilk”[tw] OR “mothers milk”[tw] | 24,723 |
| --- | --- | --- |
| #2 | “peer sharing”[tw] OR “peer-to-peer”[tw] OR “informal sharing”[tw] OR “unscreened sharing”[tw] OR “unregulated sharing”[tw] | 1,834 |
| #3 | “practice*”[tw] OR “perception*”[tw] OR “perspective*”[tw] OR “view*”[tw] OR “attitude*”[tw] OR “opinion*”[tw] OR “understanding*”[tw] OR “experience*”[tw] OR “motivation*”[tw] OR “barrier*”[tw] OR “facilitator*”[tw] | 5,165,386 |
| #4 | "Internet"[Mesh] OR “online”[tw] OR “internet-based”[tw] OR “interne*”[tw] OR “community*”[tw] OR “worldwide”[tw] | 1,347,531 |
| #5 | #1 AND #2 AND #3 AND #4 | 9 |

**Web of Science**

| #1 | TOPIC: (Human milk OR breastmilk OR mothers milk) | 67,444 |
| --- | --- | --- |
| #2 | TOPIC: (peer sharing OR peer-to-peer OR informal sharing OR unscreened sharing OR unregulated sharing) | 46,650 |
| #3 | TOPIC: (practices OR perceptions OR perspectives OR views OR attitudes OR opinions OR understanding OR experiences OR motivations OR barriers OR facilitators) | 10,542,887 |
| #4 | TOPIC: (Internet OR online OR internet-based OR communities OR worldwide) | 2,956,975 |
| #5 | #1 AND #2 AND #3 AND #4 | 19 |

**Embase (via Elsevier)**

| #1 | ‘breast milk’/exp OR ‘human milk’ OR ‘mothers milk’ | 44,696 |
| --- | --- | --- |
| #2 | ‘peer sharing’ OR ‘peer-to-peer’ OR ‘informal sharing’ OR ‘unscreened sharing’ OR ‘unregulated sharing’ | 2,617 |
| #3 | ‘practice*’ OR ‘perception*’OR ‘perspective*’ OR ‘view*’ OR ‘attitude*’ OR ‘opinion*’ OR ‘understanding*’ OR ‘barrier*’ OR ‘facilitator*’ | 7,600,328 |
| #4 | ‘internet’/exp OR ‘internet’ OR ‘online’ OR ‘internet-based’ OR ‘interne*’ OR ‘communit*’ OR ‘worldwide’ | 2,419,240 |
| #5 | #1 AND #2 AND #3 AND #4 | 12 |
