# Supplementary material for: Factors associated with informal human milk sharing among donors and recipients: A mixed-methods systematic review
Source: PLoS One. 2024 Mar 8;19(3):e0299367. doi: 10.1371/journal.pone.0299367 (PMC10923476; doi:10.1371/journal.pone.0299367)
Supplement: S3 File — (DOCX) [file pone.0299367.s003.docx]

**S3. File risk of bias assessment**

**Qualitative Research: JBI Critical Appraisal Checklist for Qualitative Research:**

Each Question is assigned **10% for Yes, 0% for No and Unclear**

**Y= Yes, N=No, U=Unclear, N/A= Not applicable**

| **Author, Year, citation number []** | **Q1** | **Q2** | **Q3** | **Q4** | **Q5** | **Q6** | **Q7** | **Q8** | **Q9** | **Q10** | **Results (%)** |
| --- | --- | --- | --- | --- | --- | --- | --- | --- | --- | --- | --- |
| Obeng et al, 2022, [59] | **Y** | **Y** | **Y** | **Y** | **Y** | **N** | **N** | **Y** | **Y** | **Y** | **80%** |
| Wagg et al, 2022, [15] | **Y** | **Y** | **Y** | **Y** | **Y** | **Y** | **N** | **Y** | **Y** | **Y** | **90%** |
| McCloskey and Karandikar, 2018, [42] | **Y** | **Y** | **Y** | **Y** | **Y** | **N** | **N** | **Y** | **Y** | **Y** | **80%** |
| McCloskey and Karandikar, 2019, [43] | **Y** | **Y** | **Y** | **Y** | **Y** | **N** | **N** | **Y** | **Y** | **Y** | **80%** |
| McNally and Spatz, 2020, [44] | **N** | **Y** | **Y** | **Y** | **Y** | **N** | **N** | **Y** | **N** | **Y** | 60% |
| Keim et al, 2014, [53] (qualitative component) | **Y** | **Y** | **Y** | **Y** | **Y** | **N** | **N** | **Y** | **Y** | **Y** | **80%** |
| Perrin et al, 2014, [54] (qualitative component) | **Y** | **Y** | **Y** | **Y** | **N** | **N** | **N** | **Y** | **Y** | **Y** | **70%** |
| Perrin et al, 2016,[45] | **Y** | **Y** | **Y** | **Y** | **Y** | **Y** | **Y** | **Y** | **Y** | **Y** | **100%** |
| Bressler et al, 2020, [46] | **Y** | **Y** | **Y** | **Y** | **Y** | **Y** | **N** | **Y** | **Y** | **Y** | **90%** |
| Gribble, 2013, [51] | **N** | **Y** | **Y** | **Y** | **Y** | **N** | **N** | **Y** | **Y** | **Y** | **70%** |
| Gribble, 2014, [50] | **N** | **Y** | **Y** | **Y** | **Y** | **N** | **N** | **Y** | **Y** | **Y** | **70%** |
| Gribble, 2014, [48] | **N** | **Y** | **Y** | **Y** | **Y** | **N** | **N** | **Y** | **Y** | **Y** | **70%** |
| Gribble, 2014, [49] | **N** | **Y** | **Y** | **Y** | **Y** | **N** | **N** | **Y** | **Y** | **Y** | **70%** |
| Gribble, 2018, [25] | **N** | **Y** | **Y** | **Y** | **Y** | **N** | **N** | **Y** | **Y** | **Y** | **70%** |
| Thorley, 2009, [58] | **N** | **Y** | **Y** | **Y** | **Y** | **N** | **N** | **Y** | **N** | **Y** | **60%** |
| Thorley, 2012, [16] | **N** | **Y** | **Y** | **Y** | **Y** | **N** | **N** | **Y** | **Y** | **Y** | **70%** |
| Wilson, 2018, [47] | **Y** | **Y** | **Y** | **Y** | **Y** | **N** | **N** | **Y** | **Y** | **Y** | **80%** |
| O’ Sullivan et al., 2016, [17] | **N** | **Y** | **Y** | **Y** | **Y** | **N** | **N** | **Y** | **Y** | **Y** | **70%** |
| O’ Sullivan et al., 2018 (qualitative component), [52] | **N** | **Y** | **Y** | **Y** | **Y** | **N** | **N** | **Y** | **Y** | **Y** | **70%** |

**Quantitative: JBI Critical Appraisal Checklist for Analytical Cross Sectional Studies:**

Each Question is assigned **12.5% for Yes, 0% for No and Unclear**

**Y= Yes, N=No, U=Unclear, N/A= Not applicable**

| **Author, Year, Citation** | **Q1** | **Q2** | **Q3** | **Q4** | **Q5** | **Q6** | **Q7** | **Q8** | **Results**  **(%)** |
| --- | --- | --- | --- | --- | --- | --- | --- | --- | --- |
| Cassar-Uhl and Liberatos, 2018, [55] | **Y** | **Y** | **Y** | **Y** | **N** | **N** | **Y** | **Y** | **75%** |
| Onat and Karakoç, 2019, [61] | **Y** | **Y** | **Y** | **Y** | **N** | **N** | **Y** | **Y** | **75%** |
| Schafer et al, 2018, [56] | **Y** | **Y** | **Y** | **Y** | **Y** | **Y** | **Y** | **Y** | **100%** |
| Palmquist and Doehler, 2016, [57] | **Y** | **Y** | **Y** | **Y** | **N** | **N** | **Y** | **Y** | **75%** |
| Perrin et al, 2014, [54] (quantitative component) | **Y** | **Y** | **Y** | **Y** | **N** | **N** | **Y** | **Y** | **75%** |
| Keim et al, 2014, [53] (quantitative component) | **Y** | **Y** | **Y** | **Y** | **Y** | **Y** | **Y** | **Y** | **100%** |
| O’ Sullivan et al., 2018, [52] (quanitiative component) | **Y** | **Y** | **Y** | **Y** | **N** | **N** | **Y** | **Y** | **75%** |

**Grey literature sources:**

**Methodological quality of grey literature according to the AACODS tool (Authority= Yes, Accuracy= Yes, Coverage= Yes, Objectivity= Yes, Date=Yes, Significance= Yes).**

Adequate quality according to the AACODS checklist, meeting all AACODS criteria for methodological quality.

AACODS checklist (Tyndall, 2010) <http://dspace.flinders.edu.au/dspace/>

**Author, Year, Citation**

**Papanicolaou, 2013, [60]**

| **AACODS** |  | **Yes** | **No** | **?** |
| --- | --- | --- | --- | --- |
| **Authority** | Identifying who is responsible for in the intellectual content.  **Individual author:**   - Associated with a reputable organization - Professional qualifications or considerable experience - Produced/published other work (grey/black) in the field? - Recognised expert, identified in other sources? - Cited by others? (use Google Scholar as a quick check) - Higher degree student under ‘expert’ supervision?   **Organisation or group:**  Is the organization reputable ( e.g. W.H.O)?  Is the organisation an authority in the field?  **In all cases:**  Does the item have a detailed reference list or bibliography? | **Y**  **Y**  **Y**  **Y**  **Y**  **Y**  **Y**  **N**  **Y**  **Y** |  |  |
| **Accuracy** | Does the item have a clearly stated aim or brief?  If so, is it met?  Does it have a stated methodology?  If so, is it adhered to?  Has it been peer-reviewed?  Has it been edited by a reputable authority?  Supported by authoritative, documented references or credible sources?  It is representative of work in the field?  If No, is it a valid counterbalance?  Is any data collection explicit and appropriate for the research?  If item is secondary material, refer to the original. Is it an accurate, unbiased interpretation or analysis? | **Y**  **Y**  **Y**  **Y**  **Y**  **Y**  **Y**  **NA** | **N**  **N** |  |
| **Coverage** | All items have parameters which define their content coverage. These limits might mean that a work refers to a particular population group, or that it excluded certain types of publication. A report could be designed to answer a particular question, or be based on statistics from a particular survey.   - Are any limits clearly stated? | **Y** |  |  |
| **Objectivity** | It is important to identify bias, particularly if it is unstated or unacknowledged.   - Opinion, expert or otherwise, is still opinion; is the authors standpoint clear? - Does the work seem to be balanced in presentation? | **Y**  **Y** |  |  |
| **Date** | For the item to inform your research, it needs to have a date that confirms relevance.   - Does the item have a clearly stated date related to content? No easily discernable date is a strong concern. - If no date is given, but can be closely ascertained, is there a valid reason for its absence? - Check the bibliography: have key contemporary material been included? | **Y**  **NA**  **Y** |  |  |
| **Significance** | There is a value judgment of the item, in the context of the relevant research area:   - Is the item meaningful (this incorporates feasibility, utility and relevance) - Does it add context? - Does it enrich or add something unique to the research? - Does it strengthen or refute a current position? - Is it integral, representative, typical? - Does it have impact? (in the same sense of influencing the work or behavior of others) | **Y**  **Y**  **Y**  **Y**  **Y**  **Y** |  |  |
