# Supplementary material for: Factors associated with informal human milk sharing among donors and recipients: A mixed-methods systematic review
Source: PLoS One. 2024 Mar 8;19(3):e0299367. doi: 10.1371/journal.pone.0299367 (PMC10923476; doi:10.1371/journal.pone.0299367)
Supplement: S4 File — (DOCX) [file pone.0299367.s004.docx]

**S4 File**

**Synthesis, integration and qualitization of motivations, enablers and barriers of donors associated with IHMS**

**Synthesis and integration of donors:**

| ***Donor motivations***  ***1: Desire to help (Altruistic motivation and value)*** | | | | |
| --- | --- | --- | --- | --- |
| **Author, Year**  **Keim *et al.,* 2014**  **Perrin *et al.,* 2016**  **Wilson, 2018**  **Thorley, 2009**  **Thorley, 2012**  **Obeng *et al.,* 2019**  **Papanicolaou, 2013**  **Gribble, 2018**  **Brezzler *et al.,* 2020**  **Gribble, 2014** | | **Findings (Sample Excerpts)**  *‘stated reason for providing milk included to help someone in need’*  *‘I mean it made me feel really good that I was able to not only supply the nutritional value to my son, but to help out another child that could use it’*  *‘I struggled to breastfeed, but then to find out that you’re not able to exclusively breastfeed your baby must feel so disheartening. So I was really excited to do that for them and also I felt so fortunate to have so much extra milk because I know that there were other mothers who were going back to work and weren’t able to breastfeed as long because they couldn’t pump much at work and then they lost their supply. I obviously had no issue with that so I felt really lucky to be able to do that for other moms’*  *‘I mean it made me feel really good that I was able to not only supply the nutritional value to my son, but to*  *help out another child that could use it’*  *‘There is nothing better to give your baby than breastmilk and to be able to give it to another baby and knowing I am helping them grow is phenomenal. It is amazing.*  *[I was] very proud to be able to help this mother and father achieve their goal of not having to give baby any milk other than human milk before her repair surgery*  *‘Happy to help a baby who needed milk for one reason or another to help a mother in need’*  *‘I was engorged. And she wasn’t producing enough to feed her child but was determined to stick to the 6 months rule’*  *‘I thought it was a great way to help someone who [was unable to] feed their baby breast milk for whatever reason…I wanted to help someone else…you are giving the baby the best start possible’*  *‘I love being able to see her son grow and to know that I contributed to his health. I would not want it any other*  *way’*  *‘We all just help one another, we felt by doing this we were giving ourselves over to Him. It was more than actually helping the child it was providing hope for the parents and family’*  *‘I had a very easy time producing breastmilk and wanted to help out'* | | |
| ***Donor motivations***  ***2: Excess/surplus milk (Altruistic motivation and value)*** | | | | |
| **O’ Sullivan et al., 2016**  **Papanicolaou, 2013**  **Thorley, 2009**  **Gribble, 2014**  **Brezzler et al., 2020**  **Wilson, 2018** | | *‘It was like 30 bags I just donated to some moms [through the Internet] because I just realized I had way too much in the freezer that I was probably not going to be able to use’*  *‘[My friend] called me and asked if she could have this, all the*  *milk that I had stockpiled, and so I gave her like hundreds of*  *ounces of milk’*  *I have been cursed and blessed with an abundant supply. If (daughter’s name) decides to skip one feeding a day, I can totally tell and it is something that I always have to work on… I remember talking to a lactation consultant at the very beginning saying you know when am I going to stop leaking all over the place? And she said oh, like when your body adjusts…I still leak all over the place.*  *‘I just gave it [reference to human milk] away because I literally had so much… We had it in everybody else’s freezer; we had to get it out of everybody else’s freezer. So once we emptied the first freezer load, then we could go collect it from the [other] houses [where] we had it [stored]’*  *‘I was honoured that she would use that from me as obviously it was bodily fluid. I was [pleased] it wouldn’t go to waste’*  *‘I was not able to throw that precious milk away’*  *'I have an oversupply'*  *‘Somebody posted that they needed milk for a sick baby in a social media group that I am a part of, many mothers had commented on the posts so I felt safe to say that I had a freezer full and I could help’*  *‘In case I do [need milk], I always have it in the back of my mind, “What if my supply drops again?” Or if something happens, and then I’m not able to produce. I always want to make sure I’m fully stocked more than enough, and then if I have the surplus and then go ahead and donate’* | | |
| ***Donor motivations***  ***3: Milk bank restrictions and objections (Resistance to commercialization and overcoming inaccessibility)*** | | | | |
| **Gribble, 2013**  **Papanicolaou, 2013**  **Gribble, 2014**  **Perrin *et al.,* 2016** | | *‘The tests, rules, and how they treated my milk put me off. I hated the idea of sending my milk somewhere and have it treated like a specimen instead of the life force it is. I hated not knowing where my milk was going. I also hated the idea of my milk being sold to another family when it didn’t need*  *to be’*  *‘I like that my milk isn’t fully processed when I share it with the family I donate to- all the good antibodies and cellular components etc are retained and able to be used instead of lost in the “cleaning”*  *process that milk bank milk undergoes’*  *‘I prefer to donate directly to the mother because I know exactly where my milk goes, I know it won’t be pasteurized and I know it’s free of charge to the mother in need’*  *‘I would not donate to a milk bank because I don’t support the way they charge exorbitant amounts of money to families who are obviously in need’*  *I did think about [donating to] a milk bank but it was out in [province in*  *Canada]…so the idea of like shipping it…Kind [of] made me think mmm no, you know, I [will] just hold on to it and then I came across the other option[reference to the online sharing of human milk utilizing a commerce-free approach].*  *It was noted that donation for some to a milk bank was not possible, practical, or acceptable to them*  *‘…they required that you go in for blood testing and do an interview and with a 12-week-old, and a full-time work schedule, I knew I didn’t have the time to go through the protocols that they would require.”*  *I’ m not sure exactly what it is, but I know that it’s very cost prohibitive for people if their insurance doesn’t cover it and even though it’s a nonprofit, I don’t believe that somebody is not getting rich off of mothers*  *donating their milk and I don’t like that. . . . Yeah, I mean the people who work there are certainly making money. Just like all of these other nonprofits that you hear of where the CEOs have ridiculous salaries.”*  *‘From what I understood it was basically just mixed with everyone else’s milk that was donated and then all pasteurized and then sold to different moms or people who wanted it for their kids. I’d heard that sometimes it was donated to preemies, but most of the time it was a money thing, which I wasn’t super pleased about.* | | |
| ***Donor motivations***  ***4: Wanted to know where the milk was going (Altruistic motivation and value)*** | | | | |
| **Perrin et al., 2016**  **Gribble, 2018** | | *‘I wanted to hear why they needed the milk a little bit more to make it more personal for me’*  *‘It's very important also so I can be sure the milk is being used as intended to be used when I donated it’* | | |
| ***Donor motivations***  ***5: Commerce free approach (Resistance to commercialization and overcoming inaccessibility)*** | | | | |
| **Gribble, 2013** | | *‘I know it’s free of charge to the mother in need’*  *‘I hated not knowing where my milk was going. I also hated the idea of my milk being sold to another family when it didn’t need to be’* | | |
| ***Donor motivations***  ***6: Intrinsic value and significance of breastmilk (Altruistic motivation and value)*** | | | | |
| **Perrin *et al.,* 2016**  **Wilson, 2018**  **Thorley, 2009**  **Papanicolaou, 2013**  **Gribble, 2014**  **Brezzler *et al.,* 2020** | | *‘I actually even still to this day, I’ll look at my son just amazed that not only did he grow inside of me, but for the first part of his life where he wasn’t eating other foods, he was solely sustained just on what my body*  *created and made for him and it’s just an awe’*  *‘the healthiest thing that [babies] can eat’*  *‘There is nothing better to give your baby than breastmilk and to be able to give it to another baby and knowing I am helping them grow is phenomenal. It is amazing.*  *The fact that they are getting the vitamins and nutrition that they need and it is ready-made …. You have it so that they can have it. That is the reason why God gave it to you*  *…. It was a rewarding experience because it was something that my body can actually do.*  *I was honoured that she would use that from me as obviously it was bodily fluid. I was [pleased] it wouldn’t go to waste.*  *I think when you are in community with breastfeeding women; I think you*  *understand the power of breast milk. So, it is something that resonates with all of us.*  *It is the healthiest for them. Healthy for mom and healthy for baby*  *‘I think that breastmilk is an extremely important part of the growth and development of a child and every parent should have the option.to provide this to their infant.’*  *More than half of respondents donated expressed breastmilk that their infant did not require, and most did so because they did not want milk to be wasted.*  *This (my milk) is one of the most powerful things that God has created…* | | |
| ***Donor motivations***  ***7: Perceived deficits of formula (Resistance to commercialization and overcoming inaccessibility)*** | | | | |
| ***Wilson, 2018***  ***Thorley, 2009***  ***Gribble, 2018*** | | *‘Why would you want to buy formula, when I have all of this frozen milk?’*  *‘The fact that they are getting the vitamins and nutrition that they need and it is ready-made’*  *‘I was very proud to be able to help this mother and father achieve their goal of not having to give baby any milk other than human milk before her repair surgery’*  *‘The baby I donate to has been able to stay on exclusively breastmilk for a long time now …. Since being on breastmilk exclusively, he is growing and thriving and experiences none of that discomfort’* | | |
| ***Donor Enablers***  ***1: Social media and internet (Synergistic enablers: uniting digital and personal connectedness)*** | | | | |
| **Papanicolaou, 2013**  **Gribble, 2018**  **Brezzler et al., 2020**  **Perrin *et al.,* 2016** | | *‘I went home and Googled share milk and I found it on Facebook. That is how I found it. So, that is how it started and I just found somebody, I found the [name of organization], so I just posted it on there’*  *‘You know we have kept in touch a little bit and if I do have extra milk, I will just email her and say next time you are in [the area], you can come and get more’*  *Internet‐based peer‐to‐peer milk sharing is facilitating not only the most personal of exchanges but also the development of real relationships*  *Somebody posted that they needed milk for a sick baby in a social media group that I am a part of, many mothers had commented on the posts so I felt safe to say that I had a freezer full and I could help.*  *It was very easy to do because all the instructions are online, I learned how to make my body make more milk, so I still had enough for my baby at home. The women were all supportive and encouraging online it was like one big family working together to care for our community.*  *I looked online to see if there were any other families in need to begin the process again.*  *‘I came across an article about milk sharing and so I kind of started, you know how they have links and stuff, clicking through the links and realizing that I could donate all this excess that I have just been dumping down the sink and I was really excited about that because it felt like such a waste, you know.”* | | |
| ***Donor Enablers***  ***2:Donor milk bank inaccessibility (Resisting commercialization and overcoming inaccessibility)*** | | | | |
| **Perrin *et al.,* 2016**  **Gribble, 2013**  **Gribble, 2014**  **Papanicolaou, 2013** | | *‘..my first thought was to go through a milk bank, but when I looked into it and the closest milk bank was like 45 minutes away and they required that you go in for blood testing and do an interview and with a 12-week-old, and a full-time work schedule, I knew I didn’t have the time to go through the protocols that they would require.”*  *“I didn’t want to deal with the hassle of packing/shipping or taking milk to a milk bank. Also, I didn’t want to have to get blood tests each*  *time like I’ve read many milk banks ask you to. It’s time consuming,” and “I would have had to pump at least 300oz within one month to qualify to donate.*  *I’m not permitted to donate to U.S. milk banks as I lived in the UK and I’m considered a risk for transmitting mad cow disease.*  *‘I had enquired into donating to a milk bank and was told that they were not taking any more donations due to financial reasons’*  *‘I wanted to donate milk to a milk bank and attempted to contact the one closest to me but never got a response’*  *“[I had milk in my freezer to donate but] I was told I would have needed to be prescreened before I started pumping.*  *A few respondents noted that donation to a milk bank was not possible, practical, or acceptable to them*  *I did think about [donating to] a milk bank but it was out in [province in*  *Canada]…so the idea of like shipping it…Kind [of] made me think mmm no, you know, I [will] just hold on to it and then I came across the other option[reference to the online sharing of human milk utilizing a commerce-free approach].* | | |
| ***Donor Enablers***  ***3: Facilitating personal connections (synergistic enablers: uniting digital and personal connectedness)*** | | | | |
| **Gribble, 2013**  **Gribble, 2018**  **Papanicolaou, 2013** | | *‘I prefer to donate directly to the mother because I know exactly where my milk goes’*  *‘I think it was very important to know why that mother needed milk. I feel like I was giving a part of myself away, so I wanted it to go to someone who truly needed it,” and “I did want to know the circumstances so that I was sure it was not going to be wasted or sold on"*  *is very important to me to make sure the milk that I have expressed will be used to provide nutrients for a baby in need.*  *It is a sacrifice and if I know the situation it is motivational for me to sit with pumps attached to me multiple times a day.*  *I do this because I know the mother and baby I donate to, and*  *truly care for them.*  *Knowing where the milk is going It's very important also so I can be sure the milk is being used as intended to be used when I donated it.*  *The baby I donate to has been able to stay on exclusively breastmilk for a long time now …. Since being on breastmilk exclusively, he is growing and thriving and experiences none of that discomfort*  *You feel a little bit like you are judging these people based on their stories and you [are] judging the need almost.*  *How do you say that one person needs it more than another?...I would probably say I, rightly or wrongly, I did look at the circumstances…I would prefer to give my milk to someone who I felt in my opinion needed it more. So, again rightly or wrongly.* | | |
| ***Donor barriers***  ***1: Healthcare related barriers (Lack of awareness and acceptance of IHMS in healthcare settings)*** | | | | |
| **Perrin *et al.,* 2016**  **Brezzler *et al.,* 2020**  **Papanicolaou, 2013** | | *Most participants reported that they received no information about milk exchange options and considerations from health care professionals*  *I didn’t even really bring it up with the doctors and they never brought it up to me. I think it was mostly me just looking stuff up on my own without any outside professional input on the matter*  *Conversations about milk exchange that were initiated by health care professionals were rare, but when they did happen, it was most often initiated by a midwife, doula, or lactation consultant.*  *The hospitals have so many rules, I don’t want to say anything wrong and mess things up where the baby won’t be able to receive any more donor milk.*  *Things are kept very private. I don’t know who else may have been involved, we just did what we believed without thinking whether we were allowed or not, like with hospital protocols*  *It still felt like I was doing some kind of back alley black market deal, like there was something, you know, wrong about it because [it] was [not] Government regulated…it does feel like you [are] some kind of secret society* | | |
| ***Donor Experiences***  ***1: Desire to assist (Selfless acts with conceptual significance)*** | | | | |
| **Papanicolaou, 2013**  **Gribble, 2018**  **Gribble, 2013**  **Perrin *et al.,* 2016**  **Wilson, 2018**  **Thorley, 2009**  **Thorley, 2012**  **Brezzler et al., 2020**  **Gribble, 2014**  **Keim et al., 2014** | | *I feel happy. I am doing something good for someone else, someone that I do [not] even know…I think those kinds of things [provide] you [with] some…soul, happiness, and peace. (Mrs. Grant)It [is] the same feeling you would get through any altruistic act, you get happiness, [a] sense of purpose [and] a sense of well-being. It makes you feel good*  *I love being able to see her son grow and to know that I contributed to his health. I would not want it any other way. [D55]- that baby got human milk, the health impact of milk on the baby*  *‘It’s gratifying to hear a story of a child in need and know that I am able to help’*  *‘I mean it made me feel really good that I was able to not only supply the nutritional value to my son, but to help out another child that could use it’*  *‘There is nothing better to give your baby than breastmilk and to be able to give it to another baby and knowing I am helping them grow is phenomenal. It is amazing.*  *‘I was very proud to be able to help this mother and father achieve their goal of not having to give baby any milk other than human milk before her repair surgery’*  *‘I was honoured that she would use that from me as obviously it was bodily fluid. I was [pleased] it wouldn’t go to waste.*  *‘Happy to help a baby who needed milk for one reason or another to help a mother in need’*  *‘We all just help one another, we felt by doing this we were giving ourselves over to Him. It was more than actually helping the child it was providing hope for the parents and family’*  *‘I had a very easy time producing breastmilk and wanted to help out'*  *‘stated reason for providing milk included to help someone in need’* | | |
| **Qualitization of quantitative data relating to donor motivations, barriers and enablers** | | | | |
| **Study** | **Quantitative findings** | | **Quantitative transformation/ qualitized data** | **Emerged Category** |
| Onat and Krackoc (2019) | 83.3% desired to help someone | | Desire to help someone | 1 Motivation- desire to help (Altruistic motivation and value) |
| Onat and Krackov (2019) | 83.3% reported excess milk production | | Almost all had an excess/surplus of milk | 2 Motivation- excess/surplus milk (Altruistic motivation and value) |
| Onat and Krackov (2019) | 68.8% did not need the milk they expressed | | The majority had an excess/surplus of milk | 2 Motivation- excess/surplus (Altruistic motivation and value) |
| Onat and Krackov (2019) | 68.8% did not pass the predonation screening | | Did not pass formal milk donation screening/milk bank inaccessibility | 3 Motivation- Milk bank restrictions and objections (Resistance to commercialization and overcoming inaccessibility) |
| Onat and Krackov (2019) | 67.1% generally asked about the reason for accepting donated milk | | Donors wanted to know the source of the recipient and reason for receiving | 4 Motivation- Wanted to know where the milk was going (Altruistic motivation and value) |
| Onat and Krackov (2019) | 100% of donors received no payment | | Commerce free approach | 5 Motivation- Commerce free approach (Resistance to commercialization and overcoming inaccessibility) |
| Onat and Krackov (2019) | 60.4% donated through the internet on milk sharing sites | | Social media and internet sites are the most common means of milk sharing | 1 Enabler- Social media and internet (Synergistic enablers: uniting digital and personal connectedness) |
| Onat and Krackov (2019) | 68.8% did not pass the predonation health screening | | The majority did not pass formal donation screening/milk bank inaccessibility enabled informal sharing | 2 Enabler- Donor milk bank inaccessibility (Resisting commercialization and overcoming inaccessibility) |
| Onat and Krackov (2019) | 83.3% deliver milk face-to-face | | Almost all meet the recipient in person enhancing personalization of the exchange | 3 Enabler- Facilitating personal connections (synergistic enablers: uniting digital and personal connectedness) |
| Palmquist and Doehler (2016) | 96.2% donate milk face-to-face | | Almost all meet the recipient in person enhancing personalization of the exchange | 3 Enabler- Facilitating personal connections (synergistic enablers: uniting digital and personal connectedness) |
| O’ Sullivan *et al* (2018) | Of those who provided their  HM to another, most, 60%provided it to a friend or other person they know | | A majority know the recipient personally | 3 Enabler- Facilitating personal connections (synergistic enablers: uniting digital and personal connectedness) |
| Onat and Krackov (2019) | 85.4% of donors stated that healthcare workers did not recommend milk donation | | The majority of healthcare workers do not support milk donation | 1 Barrier- Healthcare related barriers (Lack of awareness and acceptance of IHMS in healthcare settings) |
