# Supplementary material for: Factors associated with informal human milk sharing among donors and recipients: A mixed-methods systematic review
Source: PLoS One. 2024 Mar 8;19(3):e0299367. doi: 10.1371/journal.pone.0299367 (PMC10923476; doi:10.1371/journal.pone.0299367)
Supplement: S5 File — (DOCX) [file pone.0299367.s005.docx]

**S5 File**

**Synthesis, integration and qualitization of motivations, enablers and barriers of recipients associated with IHMS**

**Synthesis and integration of recipients:**

| ***Recipient motivations***  ***1: Physical or physiological challenges (maternal or infant factors)*** | | | | |
| --- | --- | --- | --- | --- |
| **Author, Year**  **Papanicolaou, 2013**  **Gribble, 2014**  **McCloskey and Karandikar, 2019**  **McNally and Spratz, 2020**  **O’ Sullivan, 2016**  **Perrin *et al*, 2014**  **Thorley, 2009**  **Thorley, 2012**  **Wagg *et al*, 2022** | | **Findings (Sample Excerpts)**  *‘I was domperidone. I was on the herbs. I was nursing, I was trying to use a SNS[supplemental nursing system]. I was doing everything I knew how and I still just was not producing after a number of weeks. I was just not producing a lot of milk’*  *‘I tried EVERYTHING to increase my milk supply this 3rd time [I had previously tried everything my lactation consultants told me, but while I was pregnant w/#3, I immersed myself in finding information about how to overcome and keep nursing despite [having insufficient glandular tissue]…’*  *‘I have never been able to make enough milk to sustain any of my children'*  *‘I was unable to produce enough milk for my daughter’*  *'Baby was not gaining weight, after many months of effort to gain a*  *sufficient supply of milk 1 looked into other options'*  *‘An IBCLC that I saw through the hospital we were being treated at [diagnosed me]. The diagnosis was made by assessing our history (we were in SCU at day 5 due to severe dehydration in baby) and a physical*  *examination (wide set, tubular breasts)’*  *‘I was unable to breastfeed due to a lack of production. No one has been able to identify a cause or come up with a cure'*  *‘Our... child had four tongue clippings... Mother could no longer breastfeed due to pain and nipple damage from baby Even after the medical procedures were completed, breastfeeding pain never ceased’*  *‘My daughter was born 5 weeks prematurely and had a very poor suction reflex. She was unable to latch for breastfeeding until she was 7 weeks old’*  *‘I wanted her to have the nutrition of breast milk, but wasn’t really capable to produce myself. I know it’s [inducing lactation] possible, but that looked pretty overwhelming in this situation [adoptive parent]. So, when I found out, you know, that I could have the benefits of breast milk through a donor’*  *‘The benefits were really specific for us because of [son’s*  *name] disease. I didn’t want to put a shock to his system of trying formula, of which he’s never had before and I wouldn’t know the consequences without trying it. So, formula, or anything like that wasn’t really ever an option’*  *(n=1) struggled with flat nipples, her son’s inability to latch, a subsequent tongue-tie diagnosis, (n=1) difficulty inducing lactation for adopted child, (n=1) low milk supply.*  *‘We needed my son’s weight gain to get up there so he could, you*  *know, have the energy to learn how to nurse properly, and I needed to heal because my nipples were destroyed at that point’*  *(n=2/3) of the participants in the study had low milk supply*  *Motivations described for receiving HM included experiencing a delayed onset of lactogenesis II, delivering a premature infant, taking medications that are contraindicated while at-the-breast feeding, having an inability to pump sufficient HM during maternal–infant separation, having a low milk*  *supply, assessing a problem with at-the-breast feeding, or providing HM for a fostered or adopted infant*  *69.4% of recipients cited lactation problems for the reason for seeking donor milk. ‘My daughter is 9 days old and my milk has not (and I fear, may not) come in’*  *‘We have had many issues breastfeeding and I have unfortunately*  *dried out’*  *48.5% of recipients sought milk for reasons relating to child health:*  *‘She needs breastmilk to help her immune system through these*  *next two surgeries’*  *‘Had a very hard go of it in the NICU after contracting NEC’*  *‘Because of the medication I'm taking I won't be able to*  *Breastfeed’*  *‘Cancer survivor mother in need of any amount…due to double*  *Mastectomy’*  *‘If you are desperate you would take any means that were necessary for your child. I don’t like formula milk. I had a prem, at 26 weeks. When I brought him home at over four months, another mother expressed for me’*  *‘My baby] had serious breast attachment issues for the first six months of her life. In this period we were fortunate to meet a beautiful woman who provided breast milk for us. My daughter was fed this EBM through a*  *supply line … for 4 months. At six months my daughter’s attachment improved to the point that we did not need the EBM or supply line’*  *Various reasons for seeking donor milk were cited within, including: difficulty establishing lactation, bridged a gap with initial breastfeeding challenges, maternal and infant anatomy or medical issues, insufficient milk supply*  *‘Midwife has recommended that I express every other feed but top*  *up if he is unsettled. Basically, it’s my low supply that’s causing the issues, so I’m feeling a little fragile’*  *‘So I didn’t expect it to be as hard or even not produce milk to be able*  *to feed my baby that was just a bit of a shock, that I didn’t have any milk. Yea I wasn’t expecting that’* | | |
| ***Recipient motivations***  ***2: Value of breastmilk (superiority and advantageous impact of human milk)*** | | | | |
| **Papanicolaou, 2013**  **Gribble, 2014**  **McCloskey and Karandikar, 2019**  **Wilson, 2018**  **McNally and Spratz, 2020**  **Wagg *et al*., 2022**  **Keim *et al.,* 2014** | | *‘I think when you are in community with breastfeeding women; I think you understand the power of breast milk. So, it is something that resonates with all of us’*  *‘It is the healthiest for them. Healthy for mom and healthy for baby’*  *‘I really did [not] want (daughter’s name) to suffer for what was happening to me, like you know. So, I wanted to give her the best kick at the can…that is why we wanted [her] to have breast milk’*  *‘There are lots of benefits to breastmilk. Breast milk is easier to digest for them and it is customized for them. BM changes as they grow’*  *‘I wanted [my child] to have the benefits of breastmilk'*  *‘Breastmilk truly is the best gift for a child. When I realized my body can't make it, I really wanted a better alternative than formula'*  *‘I really wanted to have all the nutritional benefits of breast milk, all of the antibodies in breast milk that sort of thing. The baby gets the benefit of having the antibodies and um, that perfect nutrition from breast milk’*  *‘The immune benefits that they get from breast milk far outweigh any potential risk. And it’s really what I wanted her to have. It’s what babies are supposed to have and it wasn’t something I could provide for her’*  *Milk sharing is “responsibility” and a good mothering “choice.*  *‘We knew at that moment the best option for her would be human milk’*  *‘breastmilk is precious and shouldn’t be wasted’*  *All of those who received donor milk reported one or more benefits to their infant (e.g., better tolerance, immunity)* | | |
| ***Recipient motivations:***  ***3: Avoidance of commercial milk formula (superiority and advantageous impact of human milk)*** | | | | |
| **Papanicolaou, 2013**  **Gribble, 2014**  **Keim *et al.,* 2014**  **McNally and Spratz, 2020**  **McCloskey and Karandikar, 2019**  **Thorley, 2009**  **Thorley, 2012**  **Wilson, 2018**  **Wagg *et al.,* 2022**  **Gribble, 2014** | | *‘I do [not] like what [is] put in formula, the artificial nature of it and just the combination of ingredients that are in there. [The ingredients] are [not] something that I would like my children to eat’*  *We just kind [of] went through the thought process of well, which is going to be more beneficial to her?...Breast milk even though it is not mine or artificial formula? So, we kind [of] were willing to accept the risks of donor milk and go that route rather than artificial, you know rather than formula*  *I know in my mind that it [is] better [reference to DHM], you know*  *especially when you look at like the World Health Organization. You know, it is best to give milk at the breast first, then second is milk from the mother, from the mother in a bottle, and then third is you know, donated milk and then it is formula…I am giving my baby something better than formula*  *‘When I realised my body can't make it, I really wanted a better alternative than formula. Knowing the inferiority of formula was a huge factor'*  *We tried every formula on the market, including prescription formulas, but none of them worked for our girls without making them in constant pain and miserable'*  *'My first child still suffers from dry, itchy skin and constipation. I believe much of that is from the formula he had to eat in his first few days of life... When I heard about this option I... got donor milk as soon as I could'*  *One woman sought milk from friends because her infant would not tolerate formula; she received 1,000–1,500 oz over 4 months from three*  *Friends.*  *One donor recipient reported that her infant never had infection in first year of life which she attributes this to using donor milk and not using formula.*  *One recipient respondents infant did not do well on formula and often got so constipated she had bloody stools. A switch to hypoallergenic formula resulted in colic, and she said ‘We knew at that moment the best option for her would be human milk’*  *‘When the moment came when I couldn’t [breastfeed], it was very, it just felt like a crisis. And so, breast milk seemed, you know, donor milk seemed like such a better option to me than formula..... from researching I was really concerned about the sugars and other things in American formula.*  *‘I mean, the number one benefit is that your baby is getting*  *breast milk as opposed to formula. I felt like there is more of*  *a risk for her to develop allergies or symptoms from taking formula. For me, it’s a last resort. I was like, she was gagging now on formula..yeah, so she wasn’t taking the formula very well.so I wanted to give breast milk and not do the formula thing. .my first daughter was formula fed and she was constantly sick .had rashes, always had, had pneumonia a couple times,had tubes put in her ears right before she turned 2—always had*  *ear infections. I was very worried that it [formula] would compromise my new daughter’s immune system. I gave one bottle of formula before finding someone to get donated milk from and my baby immediately threw it up and I knew that I could not do that to her. She’s has had no adverse reactions to any of the breast milk’*  *…If you are desperate you would take any means that were*  *necessary for your child. I don’t like formula milk…’*  *‘..So long as my child was receiving EBM from a reliable source, but not necessarily screened, I would be happy that they were receiving BM [breastmilk] over ABM...’*  *A responded received donor expressed milk to meet her desire of avoiding formula milk*  *‘It was kind of a personal choice that I would rather use human breastmilk than formula. And that is my own choice to do that as far as she is concerned … I am not expecting to put any formula in the home. Not samples. No bottles, nothing’*  *One recipient protects her children from factory contamination and from cold, profit-driven, patriarchal biomedicine. Her thinking is progressive instead, rejecting anything too commercial (such as formula) or needlessly interventionist (such as milk banks)*  *‘….we haven’t got to use that formula, he won’t get belly ache any longer because he was really struggling with constipation and I was like this formula isn’t working and he can have breastmilk, I can keep*  *breastfeeding him…’*  *Recipient respondents identified a large number of risks associated with*  *formula feeding which played a role in the decision to use peer-milk sharing* | | |
| ***Recipient motivations***  ***4: Maternal mental health/well-being (superiority and advantageous impact of human milk)*** | | | | |
| **Gribble, 2014**  **McCloskey and Karandikar, 2019**  **Wagg *et al.,* 2022** | | *‘It also made me feel depressed every time I had to supplement him and I hated seeing that can of GoodStart [infantformula] sitting on the table. I knew there had to be something better’*  *So when I found this [milk sharing] was available, it really gave me piece of mind. Like I can still give her that quality without stressing myself out, you know?*  *‘I felt like I could breathe. There’s this stigma of once you use the formula that it was kind of a slippery slope and I didn’t want to go down that. It was just a stress relief 100% that I had that option’*  *‘It’s been, it’s helped me so much .And after I received donated milk it was easier on me because stress does affect, you know, your milk supply. So I was a little more relaxed. I started noticing that my supply was being affected in a positive way’*  *‘Looking back I probably was not in a great mental state, honestly breast milk sharing alleviated some of the stress of feeding her. I think if I had switched her to formula or started supplementing with formula, I was really worried about losing my supply completely if I did that. So, I think*  *that would’ve really depressed me and made me feel like I failed her, at least at the beginning it’s hard to say what would’ve happened if I hadn’t. But, I think it was positive’*  *Many participants suggested that utilizing donated human milk may have supported positive mental health and even protected them from and/or alleviated some symptoms of PPD and anxiety.*  *All participants reported a reduction in perceived stress as a result of using donated breast milk from a peer*  *‘Just amazed, just... I was amazed that people do that for other people, amazed about it but also gutted that I didn’t know about it for my other children because it would have made such a difference to how I felt after. Because I was diagnosed with postnatal depression, I think it would have really helped me feel not as low as I did in those times, because I would have still given them breastmilk which to me is preference over formula’*  *‘…Um, it saved my sanity and my mental health, yea I was overjoyed completely overjoyed, overwhelmed, overwhelmed by somebody else’s kindness, and it means my baby is healthy and thriving and happy’.* | | |
| ***Recipient motivations***  ***5: Donor milk bank related (Human milk bank influences)*** | | | | |
| **Gribble, 2014**  **Gribble, 2013**  **McCloskey and Karandikar, 2018**  **McNally and Spratz, 2020**  **Wilson, 2018**  **Wagg *et al.,* 2022** | | *‘…I didn't make enough milk and the milk banks said they could*  *not sell me any milk [it was too expensive anyway]’*  *‘I contacted a milk bank, but they stopped responding to me after my first email. My daughter does not have a ‘medical need’’.*  *‘I had looked into obtaining milk from a human milk bank . . . but it*  *seemed to be only available for preterm or sick babies and even if I could obtain it, it was very expensive’*  *‘I knew that we could not afford milk banks as I had looked into this with my preemie [previous child] and at $4/oz that was way beyond what we could afford’*  *‘There is no local milk bank in our area’*  *‘I looked into and I think the idea is good, but in practice . . . they [milk bank] told me that my daughter was too old to receive milk. . . . They were charging about 4 dollars. . . . Milk banks also tend to pasteurize the milk and that kills a lot of the good proteins that are in it’*  *‘I’m in a very rural area and the closest milk bank is approximately*  *5 hours away’*  *‘I mean I had heard of it [milk bank] before. But . . . it [peer-to-peer HM sharing] made me less hesitant than a person that I didn’t know’*  *And so also for a short period of time, I bought some milk . . . from the milk bank. . . . But, it was like 20 dollars for 4 ounces. So I didn’t do that for very long because it was very quickly, not sustainable.*  *‘Cost of human milk banks and the difficulty of inducing lactation, however, made these two options unfeasible’*  *One participant rejected anything too commercial (such as formula) or needlessly interventionist (such as milk banks)*  *‘I’m using donor milk from a local friend but running low so will probably need to use formula again soon bit it made him horribly constipated before. We have started the process of applying to the milk bank, but I doubt I will get funding or accepted as we are low on the order of need’* | | |
| ***Recipient motivators***  ***6: Return to work/ School (Maternal of infant factors)*** | | | | |
| **Gribble, 2014**  **Perrin et al, 2014** | | *‘I returned to work when my daughter was 7 months and I was not able*  *to pump enough during the day. When I am with her I am*  *able to feed fine. It was just that I am not responding to the*  *pump'*  *‘I was not able to pump more than 8 minutes while at work 2x a day’* | | |
| ***Recipient enablers***  ***1:Social media/ Internet (Digital connections and transparency)*** | | | | |
| **Gribble, 2018**  **Papanicolaou, 2013**  **Wagg *et al.,* 2022**  **Gribble, 2014** | | *‘We catch up every now and again and keep up with how our kids are going. Sometimes chat on facebook’*  *‘It is funny because the Internet brought us together but yet we are doing*  *something [reference to sharing human milk] that you know people have done since the beginning of time. So, it is kind [of] a neat full circle that happened’*  *‘It is easy, you can access it [reference to organization’s Facebook site] from virtually anywhere that there [is] a signal’*  *‘It [is] hard to find people, it is a very small subset of people who would be willing to do that [reference to sharing human milk] and I think that the Internet really allows you to connect with those few people who are out there’*  *‘I suppose it’s, it is social media that has made it more, more like, accessible to me, I thought in our area it was just for NICU and new babies … because it’s there all the time, someone to encourage to say*  *keep going you’re doing well, which in the middle of the night is vital’*  *‘I made connections with my donors and got to know them on a personal level before accepting milk, the more I messaged them and talked to them the more comfortable I became’* | | |
| ***Recipient enablers:***  ***2: Trust and transparency (Digital connections and transparency)*** | | | | |
| **McCloskey and Karandikar, 2018**  **Gribble, 2014**  **Gribble, 2018**  **Thorley, 2009**  **Thorley, 2012**  **Wagg *et al.,* 2022**  **Papanicolaou, 2013**  **Wilson, 2018** | | *‘Um, so they’re kind of unspoken rules . . . they often will say, “I don’t smoke. I only take vitamins. You know, I don’t drink milk. This is lactose free or whatever.” Um, and even when I, I’m like . . . “I hate to ask this because I’m begging for your milk, but are you healthy?” [laughs] . . . everybody’s been, “no, it’s totally fine. You need to ask that. You’re the mother. You need to be protected’*  *I trusted her. She was already an acquaintance of the family. Her son she*  *was still breastfeeding was very healthy. She knew my midwife*  *‘The amount of information they were willing to provide me regarding their health and the health of their child’*  *‘They are all breastfeeding moms in our community, who seem very sincere in wanting to donate their extra milk to help another mom/baby. Many have disclosed freely if they are taking any kind of medications or even if they drink a glass of wine occasionally they seem very honest’*  *‘I got to know the moms and got copies of their bloodwork results’*  *‘I made connections with my donors and got to know them on a personal level before accepting milk .the more I messaged them and talked to them the more comfortable I became’*  *The reasons for rejection (in order of frequency) included that: the donor was consuming a drug or medication; the recipient had a bad feeling about the situation; there was difficulty communicating with the potential donor or arranging milk pick up*  *‘I think it's important if only so you can build trust with someone you are consistently getting milk from’*  *‘As long as we have a consensual agreement – if some kind of crisis. I would prefer to know beforehand and have some kind of arrangement. [I wouldn’t] if they are not in my friendship circle, a drug-taker, imbibing something’*  *Although no formal screening was conducted, it was clear that the*  *women informally screened those with whom they shared their milk.*  *Donors would be acceptable ‘if I or my midwife knew their medical status’, so ‘where [the milk] is not pasteurized, I would want that personal connection’*  *‘We talked before and she was very open about everything that she had been through um how many children she had and the whole background which made me feel happier about doing, about accepting the milk. I suppose if somebody wasn’t very open or preferred not to discuss certain areas that I did ask about, like smoking and drinking. Because those*  *things would put me off because I don’t do those things, or if they were on lots of medications, I think at the start that would have put me off’*  *‘I do ask questions of each donor. You know, did they receive regular prenatal screening, including HIV [human immunodeficiency virus]. I know that you [cannot] possibly be screened for everything that can be but [I ask] what is their general health and if they take any medications’*  *‘I want to know what their lifestyle is like and what, do they smoke and do they drink? And even not smoking and drinking but do they eat healthy?’*  *‘I think the criteria for me would revolve around their lifestyle, you know health, diet, choices, any consumption of alcohol, drugs [or] cigarettes’*  *You feel awkward asking, you know, are you on any medications and do you have health issues that you are aware of? And you know, but these are questions that you need to ask. Do you drink alcohol? Do you smoke? …You know you feel intrusive asking those questions especially for someone who is willing to give you something.*  *‘If we went to pick it [up] [reference to DHM], we would often sit and chat for a while…then I would, I offer for them to see [daughter’s name] and stuff, just to see where it is going to’*  *‘I guess there is a little bit of comfort in knowing that if a mom is providing it to her own child that chances are, you know it [is] okay for my child as well’*  *‘I thought, okay, if someone is taking the time to pump, they are probably a mom like me. They are not someone who is trying to hurt my kid and spike the milk and all of this kind of stuff. So I was like okay. I went on and I put in my request that I was looking for it’* | | |
| **Recipient *enablers:***  ***3: Distinct healthcare professionals (Healthcare professional facilitation of IHMS)*** | | | | |
| **McCloskey and Karandikar, 2018**  **O’ Sullivan *et al.,* 2016**  **McNally and Spratz, 2020** | | *Some mothers reported that lactation consultants, nurses, and other professionals supported and/or helped facilitate HM sharing*  *from a peer*  *I think that the fact that the nurse at the hospital supported it so much and helped me make those connections really helped increase my positive perspective on the milk sharing . . . the nurse at the hospital really helped make it feel like this is normal*  *‘My midwives just kind of jumped to it and were like, “look, we have . . . other mums that we know well and, we know their health history, so they are good and safe and, you know, we’ll ask them” and they went and got the milk for me ’cause I literally, had just given birth’*  *‘He’s [child’s pediatrician] pretty open-minded so he’s been actually helpful with it. Some of his patients have donated’*  *‘We’ve written you guys a temporary policy so that you guys are fine [and can use the donor milk in the hospital]. And we are going to rewrite our hospital policy so that this doesn’t happen again’*  *‘So the lactation consultant was at our house and so she called the*  *midwife that knew the donor and, and then the midwife just immediately called the donor, and within . . . 10 minutes, they called back and said, “Yeah, she’s got tons of milk; just come over.”*  *At her final meeting with a lactation consultant, a recipient was introduced to informal milk sharing.* | | |
| ***Recipient barriers***  ***1: Healthcare related barriers (Professional and logistical implications)*** | | | | |
| **McCloskey and Karandikar, 2018**  **McNally and Spratz, 2020** | | *‘I’d finally had the guts to ask her [lactation consultant] . . . what do you think about donated breast milk from moms in a Facebook community? And she’s like, “Well, legally I can’t, um tell you what to do or, or I can’t tell you to do that because that’s not what we’re supposed to suggest it to people’*  *You don’t really see that [education about donor milk] as much just in the hospital world of “here’s formula. Here’s formula.” There’s no suggestions of like, “hey, what about milk sharing?” . . . So that would be the number one thing I’d change, just knowing about it before I struggled’*  *‘Many mothers reported that formal and informal rules prohibit healthcare professionals and/or organizations from recommending or permitting the use of HM from a peer ‘And, actually, we, in the hospital, we were asked not [emphasis] to give the donated milk because they treat milk a medicine. And they were saying they can’t guarantee where it was from, who the donor was, and what we were giving him’*  *One such challenge was the lack of support from a pediatrician, who the couple felt tried to insinuate that a donor recipient was making the wrong decision*  *[Our pediatrician] was completely in the dark. I was schooling her…She had absolutely no idea about donor milk* | | |
| ***Recipient barriers***  ***2: Perceived donor milk supply (Professional and logistical implications)*** | | | | |
| **Gribble, 2018**  **McCloskey and Karandikar, 2019**  **McNally and Spratz, 2020**  **Wagg *et al.,* 2022** | | *Other negative repercussions were identified by recipients*  *infrequently included worrying about running low on milk*  *‘When we get a little bit low, like right now, I am a little bit stressed out. I know we have formula as a backup, but it helps to have a lot of extra*  *breast milk in the deep freezer just so we are set’*  *One participant reported that her biggest challenge was running dangerously low on her donor milk supply*  *‘I’m using donor milk from a local friend but running low so will probably need to use formula again soon bit it made him horribly constipated before. We have started the process of applying to the milk*  *bank, but I doubt I will get funding or accepted as we are low on the order of need’* | | |
| ***Recipient barriers***  ***3: Time-related barriers (Professional and logistical implications)*** | | | | |
| **Papanicolaou, 2013**  **McCloskey and Karandikar, 2018**  **McCloskey and Karandikar, 2019**  **McNally and Spratz, 2020** | | *‘….We [are] already traveling back and forth, but we do make a point to stop and sometimes we go out of our way, sometimes we have gone significantly out of our way, an hour, an hour and a half outside of our way in order to get [the DHM]’*  *‘It is exhausting. I’m not going to lie [laughs]. I’ve driven over an hour,*  *especially when my little girl doesn’t like the car seat, it takes dedication to meet people and work our schedules around their schedules and around work and whatever’*  *‘One of my donors in the beginning was 45 minutes to drive one way. So that’s a long time for a little baby to be in the car on a regular basis. Even for me as a new mom, I was so tired sometimes. So the driving is an aspect too’*  *One donor recipient received milk from about 10 donors and drove up to 2 hours to obtain it* | | |
| ***Recipient experiences:***  ***1: Grief and disappointment (the emotive and transformative power of IHMS)*** | | | | |
| **Papanicolaou, 2013**  **Perrin et al., 2014**  **Thorley, 2012**  **Wagg et al., 2022**  **McCloskey and Karandikar, 2019**  **Gribble, 2014** | | *‘There was a deep sadness for me. I had to grieve the loss because it was a loss for me. I had always anticipated that I would nurse all of my children’*  *“We have had many issues breastfeeding and I have unfortunately dried out”*  *“If you are desperate you would take any means that were necessary for your child”*  *“…Basically, it’s my low supply that’s causing the issues, so I am feeling a little fragile..”*  *So I didn’t expect it to be as hard or even not produce milk to be able to feed my baby that was just a bit of a shock, that I didn’t have any milk. Yea I wasn’t expecting that.*  *“When the moment came when I couldn’t [breastfeed], it was very, it just felt like a crisis…”*  *“It also made me feel depressed every time I had to supplement him….”* | | |
| ***Recipient experiences:***  ***2: Reduced stress and increased comfort (the emotive and transformative power of IHMS)*** | | | | |
| **McCloskey and Karandikar, 2019**  **Wagg et al., 2022** | | *“So when I found this [milk sharing] was available, it really gave me piece of mind. Like I can still give her that quality without stressing myself out, you know?”*  *“I felt like I could breathe…. It was just a stress relief 100% that I had that option”*  *“Looking back I probably was not in a great mental state, honestly breast milk sharing alleviated some of the stress of feeding her”*  *All participants reported a reduction in perceived stress as a result of using donated breast milk from a peer*  *“… but also gutted that I didn’t know about it for my other children because it would of made such a difference to how I felt after. Because I was diagnosed with postnatal depression I think it would have really helped me feel not as low as I did in those times….”*  *“….Um, it saved my sanity and my mental health….”* | | |
| **Qualitization of quantitative data relating to donor motivations, barriers, enablers** | | | | |
| **Study** | **Quantitative findings** | | **Quantitative transformation/ qualitized data** | **Emerged Category** |
| Onat and Krackoc (2019) | Milk insufficiency was reported as the primary cause of feeding an infant with donor milk, and 87.5% of the recipients participated in milk sharing because of this reason | | Maternal milk insufficiency was the predominant reason for engaging in informal human milk sharing | Motivation 1- Physical or physiological challenged (maternal and infant factors) |
| Onat and Krackoc (2019) | The other most common reason related to milk sharing were to  prevent the use of formula at the rate of 62.5% | | Preventing the use of commercial milk formula was a significant reason for engaging in informal milk sharing | Motivation 3-Avoidance of commercial milk formula (superiority and advantageous impact of human milk) |
| Onat and Krackoc (2019) | Donor milk is the most natural way to feed an infant when  mother’s own milk is absent at the rate of 62.5% | | Breastmilk is viewed as the optimal means of providing milk to infants when mothers milk is unavailable | Motivation 2- Value of breastmilk (superiority and advantageous impact of human milk) |
| Onat and Krackoc (2019) | 62.5% found donor milk through sharing  sites on the Internet | | Social media is the most common means for recipients to source donor milk and enables the practice | Enabler 1- Social media/Internet (digital connections and transparency) |
| Cassar-Uhl and Liberatos (2018) | 57.2% of recipients sourced donor milk from a milk sharing organization  44.2% of recipients sourced donor from online social network | | Social media is the most common means for recipients to source donor milk and enables the practice | Enabler 1- Social media/Internet (digital connections and transparency) |
| Onat and Krackoc (2019) | 81.3% asked their donor mothers about  cigarette and alcohol use, 75% queried the drugs used continuously, and 62.5% inquired about compliance with hygiene rules while expressing milk. Recipients were concerned about: 50%-  disease transmission, 68.8%-potential drug, cigarette, and alcohol use  37.7%-exposure to environmental toxins. 37.5% side effects or disease transmission, and 62.5%- about the risks pertaining to hygiene, storage,  and transfer conditions  75% of the recipient mothers and  85.4% of the donor mothers did not enter into any written  agreement | | The majority of recipients undertook informal screening by means of asking questions relating to cigarette, alcohol, drugs and hygiene practices with milk expression.  Concerns relating to hygiene practices, disease transmission, potential drug, cigarette or alcohol use was common among recipients.  Almost all milk sharers did not enter into a written agreement for the exchange. | Enabler 2- Trust and transparency (digital connections and transparency) |
| Cassar-Uhl and Liberatos (2018) | 83.8% of women who received donor milk reported the reason for choosing this option was because it was the healthiest option | | Most women who chose donor milk as a supplemental choice did so because it was the healthiest option | Motivator 2- Value of breastmilk (Superiority and advantageous impact of human milk) |
| Cassar-Uhl and Liberatos (2018) | 45% of women who engaged in donor milk sharing because they were concerned of the health risks of other options | | Almost half of participants who engaged in informal milk sharing were concerned about the health risks with other choices | Motivator 3- Avoidance of commercial milk formula (Superiority and advantageous impact of human milk) |
| Palmquist and Doehler (2016) | 57.8% of recipients sourced donor milk due to an infant related issue | | A majority of recipients sought donor milk due to an infant related issue | Motivator 1- Physical or physiological challenges (Maternal or infant factors) |
| Schafer *et al.,* 2018 | 68.2% reported a low milk supply | | Maternal milk insufficiency was the predominant reason for engaging in informal human milk sharing | Motivator 1- Physical or physiological challenges (Maternal or infant factors) |
| Schafer *et al.,* 2018 | Emotions related to recipients decisions to feed donor milk considered the positive and negative affect. Positive affect reported: Empowered 3.19 (1.10), 0.00–4.00  Relief 3.78 (0.54), 0.00–4.00  Happy 3.60 (0.73), 0.00–4.00  Confident 3.46 (0.89), 0.00–4.00  Informed 3.60 (0.65), 0.00–4.00  *Mean positive affect 3.53 (0.61), 1.20–4.00*  Negative affect reported:  Sad  1.01 (1.07), 0.00–4.00  Inadequate  0.86 (0.97), 0.00–4.00  Anxious  0.86 (0.97), 0.00–4.00  Confused  0.29 (0.60), 0.00–3.00  *Mean negative affect*  *0.96 (0.80), 0.00–3.75*  *Netpositive (N = 205, outcome)*  *2.57 (1.23), −2.15‐4.00* | | Recipients generally felt positive emotions relating to their decision to feed donor human milk including: relief, happy, informed and empowered. Respondents rarely felt negative emotions relating to their decision to feed donor milk. | Motivator 4- Maternal mental health/well-being (Superiority and advantageous impact of human milk) |
| Palmquist and Doehler (2016) | 64.1% of recipients received milk from online acquaintance that they met in person  36.4% of recipients received milk from online acquaintance that they had not met in person | | Online platforms enable the practice of informal human milk sharing. The majority of recipients use online platforms as a means of facilitating the sharing of milk. The most predominant category is receiving milk from an online acquaintance that they have met in person. | Enabler 1- Social media/Internet (Digital connections and transparency) |
| Cassar-Uhl and Liberatos (2018) | Recipients reported the source of where donor milk was identified. The category with the lowest responses was healthcare professional. Results: (n=138, total)  Medical professional 25/18.1%  Breastfeeding specialist 28/20.3%  Breastfeeding support group 11/8.0 | | One in five reported finding their donors through medical/  breastfeeding professionals or breastfeeding support groups. This was the lowest scoring category but implies some healthcare professionals enable milk sharing. | Enabler 3- Distinct healthcare professionals (Healthcare professional facilitation of IHMS) |
| Perrin et al (2014) | 18/7.7% of respondents requested donor milk due to work/school commitments | | The return to employment/ school is a motivator for recipients to engage in IHMS | Motivator 6- Return to school or work (Maternal or infant factors) |
