# Supplementary material for: Factors associated with informal human milk sharing among donors and recipients: A mixed-methods systematic review
Source: PLoS One. 2024 Mar 8;19(3):e0299367. doi: 10.1371/journal.pone.0299367 (PMC10923476; doi:10.1371/journal.pone.0299367)
Supplement: S1 Table — (DOCX) [file pone.0299367.s006.docx]

**S1. Table Characteristics of Included Studies**

| **Author, Year, Country/Region(s), Citation []** | **Study Aim** | **Participants** | **Data collection method (DC), Data analysis technique (DA)** | **Quality Appraisal Outcome** |
| --- | --- | --- | --- | --- |
| ***Qualitative studies*** | | | | |
| Obeng et al, 2022, Ghana, [59] | to evaluate breastfeeding prevalence and human milk sharing practices  among Ghanaian women. | (100%, n=1050)  (8%, n=86) Donors | (DC) Online survey  (DA) descriptive qualitative analysis. | Included |
| Wagg et al, 2022, UK, [15] | exploratory study observing the attitudes, thoughts, and feelings of one mother seeking human donor milk through online groups. | (100%, n=1)  Recipient | (DC) Recorded notes by participant and interview  (DA) narrative description and pattern matching analysis | Included |
| McCloskey and Karandikar, 2018, USA and Canada, [42] | experiences of mothers who have received donated human milk from a peer. | (100%, n=20)  Recipients | (DC) Interviews  (DA) Attribute and magnitude coding (Saldana, 2015) and iterative coding (Strauss and Corbin, 1990) | Included |
| McCloskey and Karandikar, 2019, USA and Canada, [61] | Recipients motivations for participating in peer-to-peer breast milk exchange, as well as the connection between receiving donated milk and postpartum stress and mental health in mothers. | (100%, n=20) Recipients | (DC) Interviews  (DA) Iterative coding (Strauss and Corbin, 1990) | Included |
| McNally and Spratz 2020, USA, [44] | Challenges, successes, motivations, barriers of informal milk sharing among recipients. | (100%, n=3)  Recipients | (DC) Interviews  (DA) Thematic analysis (Braun and Clarke 2006) | Included |
| Perrin et al, 2016, USA, [54] | An exploration of the experiences of peer-to-peer milk sharers | (100%, n=27)  Donors | (DC) Interviews  (DA) Iterative coding | Included |
| Bressler et al, 2020, USA, [46] | An exploration of the lived experience of informal donation of Ultra-Orthodox mothers | (100%, n=14) Donors | (DC) Interviews  (DA) Qualitative phenomenological analysis (Edmund Husserl’s framework and Colaizzi’s method of analysis) | Included |
| Wilson, 2018, USA, [47] | An exploration of how breastmilk sharers counter risk narratives | (100%, n=58)  Donors and Recipients | (DC) Interviews  (DA) Grounded theory data analysis and coding (Glaser and Strauss 1967 and Strauss and Corbin 1997) | Included |
| Gribble, 2014a, USA, Europe, Oceania, Asia  *‘I’m happy to help’* [48] | To examine the beliefs and practices of individuals involved in peer-to-peer milk sharing | (100%, n=97)  Donors | (DC) Survey  (DA) Descriptive statistics and qualitative content analysis | Included |
| Gribble, 2014b, *‘A better alternative’* Australia, Canada, Malaysia, New Zealand, USA, [49] | Exploration of the processes, beliefs and practices of women who used online peer-to-peer milk sharing | (100%, n= 41) Recipients | (DC) Interviews  (DA) Descriptive statistics and qualitative content analysis | Included |
| Papanicilaou, 2013, Canada, [60] | To explore the description of sharing human milk  utilizing an online commerce-free approach. | (100%, n=13)  Recipient (n=1)  Donors (n=9)  Recipients and Donors (n=3) | (DC) Interviews  (DA) Qualitative content analysis | Included |
| Gribble, 2013, USA, Europe, Oceania, Asia, [51] | To examine women’s perceptions of peer-to-peer milk sharing and milk banking | (100%, n=139)  Donors (n=98)  Recipients (n=41) | (DC) Online survey  (DA) Descriptive statistics and qualitative content analysis | Included |
| Gribble, 2014, USA, Europe, Oceania and Asia ‘*perception and management of risk’* [50] | To explore the perception and management of risks of peer-to-peer milk sharing | (100%, 138)  Donors (n=97)  Recipients (n=41) | (DC) Online survey  (DA) Descriptive statistics and qualitative content analysis | Included |
| Gribble, 2018, USA, Europe, Oceania, Asia, [25] | To explore relationships between individuals involved in internet-facilitated peer-to-peer milk sharing | (100%, n=138)  Donors (n=97)  Recipients (n=41) | (DC) Online survey  (DA) Descriptive statistics and qualitative content analysis | Included |
| Thorley, 2009, Australia, [58] | To explore the experiences of mothers involved in the sharing of breastmilk or shared breastfeeding | (100%, n=43)  Donors and Recipients | (DC) Interview (e-mail or telephone)  (DA) Unreported | Included |
| Thorley, 2012, India, Australia, Indonesia, USA, Columbia, Canada, Lebanon, Netherlands, [16] | To observe the practices of women who shared breastfeeding in a number of cultures | (100%, n=23)  Donors and Recipients | (DC) Interviews (e-mail or telephone)  (DA) Thematic analysis | Included |
| O’ Sullivan et al., 2016, USA, [17] | To explore the experiences of and attitudes towards human milk sharing among women with experience of human milk feeding, breast bump use | (100, n=41)  Mothers | (DC) Interviews  (DA) Inductive analysis | Included |
| **Citation (Year), Country/Region** | **Study Aim** | **Participants** | **Data collection method (DC), Data analysis technique (DA)** | **Quality Appraisal Outcome** |
| ***Mixed method studies*** | | | | |
| O’ Sullivan et al 2018, USA, [52] | To describe the attitudes towards human milk sharing and selling the prevalence of human milk sharing and selling in a sample of US Mothers | *Quantitative*  (100%, n=456) Postnatal Mothers  *Qualitative*  (100%, n=41) | Quantitative component:  (DC) Online questionnaire  (DA) Descriptive statistics  Qualitative component:  (DC) Interviews (DA) Content analysis and coding | Included |
| Keim et al., 2014, USA, [53] | To examine the prevalence, maternal and infant characteristics of milk sharing. To examine the awareness, consideration, participation and reasons for milk sharing | *Quantitative* (100%, n=499) Postnatal women  *Qualitative* (100%, n=11)  Recipients and Donors | Quantitative component:  (DC) Survey of women (n=499)  (DA) Univariate, bivariate, and exact logistic regression.  Qualitative component:  (DC) Interviews  (DA) Unreported | Included |
| Perrin et al., 2014, USA, [54] | To describe the size and activity of online milk sharing communities | *Quantitative* (100%, n=2) Commerce-free milk sharing online networks in USA  *Qualitative*  (100%, n=954) milk sharing donors and recipients | Quantitative component:  (DC) Counting of Facebook “likes” from two commerce-free milk sharing networks (Human milk for Human Babies HM4HB Facebook communities in 50 states) and Eats on Feets (EOF) Facebook communities in 47 states).  (DA) Descriptive statistics  (DC) Observation of Facebook posts  (DA) Content analysis and coding | Included |
| **Citation (Year), Country/Region** | **Study Aim** | **Participants** | **Data collection method (DC), Data analysis technique (DA)** | **Quality**  **Appraisal Outcome** |
| ***Quantitative Studies*** | | | | |
| Cassar-Uhl and Liberatos 2018, USA, Europe, South Pacific, Asia/Middle East, [55] | To assess the prevalence and experiences of shared milk use among breastfeeding mothers with insufficient milk supply and compare shared milk users with non-uses. | (100%, n=475)  Milk sharers (n=138) and non-milk sharers (n=337) | (DC) Internet based survey  (DA) Descriptive statistics | Included |
| Onat and Karakoç 2019, Turkey, [61] | To record the religious concerned views and attitudes towards breastmilk sharing and risk reduction strategies of mothers | (100%, n=435)  Donors (n=48)  Recipients (n=16)  Non-milk sharers (n=371) | (DC) Online questionnaire  (DA) Descriptive statistics | Included |
| Schafer et al, 2018, USA, [56] | To explore factors associated with emotional responses of recipients of shared breastmilk | (100%, n=205) Recipients | (DC) Online cross-sectional survey  (DA) Descriptive and inferential statistics | Included |
| Palmquist and Doehler ,2016, USA, [57] | To describe human milk sharing practices in the U.S | (100%, n=867)  Donors (n=661)  Recipients (n=206) | (DC) Online survey  (DA) Descriptive and inferential statistics | Included |
